# Development of a variable ground pressure tracked chassis for soil compaction mitigation

**DOI:** 10.1371/journal.pone.0359168

**Published:** 2026-11-05

**Authors:** Peichen Chu, Zhao Ding, Zhan Su, Meng Fang, Zhong Tang

**Affiliations:** 1 School of Agricultural Engineering, Jiangsu University, Zhenjiang, China; 2 Key Laboratory of Crop Harvesting Equipment Technology of Zhejiang Province, Jinhua University of Vocational Technology, Jinhua, China; Beijing Institute of Technology, CHINA

## Abstract

Traditional tracked agricultural machinery with a fixed contact geometry struggles to balance high maneuverability on hard pavements and low soil compaction in soft paddies. To solve this problem, this paper proposes an active variable ground pressure tracked chassis system. The effective ground contact length of the track is adjusted by distributed electric cylinders and a multi-stage linkage mechanism. To evaluate its performance, we conducted kinematic modeling, finite element analysis (FEA), and field tests. First, a planar kinematic model was established to describe the relationship between the cylinder stroke and the track geometry. Second, an internal load transfer path method was used in the FEA post-processing to reduce numerical errors from the track lugs, showing that the low-pressure mode reduces the mean compressive stress from 0.03 MPa to 0.008 MPa. Finally, soil bin tests with a physical prototype were carried out. The results indicate that the low-pressure mode decreases the peak subsoil stress from 93 kPa to 63 kPa at low speeds. In contrast, the regular mode with a shorter track length on hard surfaces reduces steering resistance. This study provides a practical design method for adaptable agricultural tracked vehicles.

## 1. Introduction

With the development of agricultural mechanization, tracked vehicles have been widely used in paddy fields and hilly areas due to their large contact area and traction [1–3]. Consequently, it has become the core power machinery for agricultural operations in the paddy fields and hilly and mountainous areas of Southern China [4–7]. However, traditional tracked chassis with fixed configurations often cause soil compaction and surface sinkage during frequent field operations [8–10]. Such irreversible damage to the physical structure of the underlying soil [11–15] not only deteriorates the physicochemical properties of the cultivated layer [16–18], given that the dynamic load of the tracks can penetrate the soil to a depth of at least 30 cm [19], but also severely restricts the continuous passability of heavy agricultural machinery in soft and wet environments [11,12]. Furthermore, complex and variable agricultural environments subject the ground contact characteristics of tracked chassis to a long-standing engineering trade-off between sinkage prevention and high maneuverability. According to terramechanics theory, significant step-like differences exist in the ultimate bearing capacities of various ground surfaces 160 kPa for general soft soil versus 9.8 kPa for bogs. When operating in soft and wet farmlands, the chassis must maximize its effective ground contact length to achieve low-pressure protection and prevent the machine from sinking into the mud [20,21]. Conversely, during transit on hard pavements or when crossing ridges, a lengthy ground contact track induces substantial lateral slip resistance and power loss. In summary, traditional tracked chassis featuring a single, fixed configuration can no longer overcome the inherent physical barrier between achieving low-pressure high passability on soft surfaces and low-resistance high maneuverability on hard pavements [1,22].

In the field of agricultural machinery, to meet the operational requirements in complex terrains such as hilly and mountainous areas, researchers have proposed various attitude adjustable tracked chassis schemes [4, 23]. For example, Hu, J. proposed an omnidirectional attitude-regulating chassis based on a five-bar mechanism, which was specifically designed for tracked combine harvesters. The feasibility of this design in achieving whole-machine lifting, backward pitching, and lateral rolling was verified through coupled rigid-flexible body simulations, effectively enhancing the stability and anti-rollover capability on rugged terrains [24]. Hu, J.P. et al. developed a four-point adjustable lifting tracked chassis and constructed an adaptive leveling system. By perceiving the body attitude in real time and driving the actuators for compensation, the quality and efficiency of harvesting operations were significantly improved [25,26]. Furthermore, Ding, R. et al. proposed a tracked agricultural chassis structure with a dual-layer or triple-layer frame. By employing servo electric cylinders as driving elements in conjunction with an adaptive sliding mode control algorithm, a high-precision omnidirectional leveling function was achieved [4]. Additionally, to reduce soil compaction, researchers have analyzed the impact of tracks on the soil [27,28]. To mitigate the risk of paddy soil hardening caused during steering operations, a forward-and-reverse steering gearbox developed by Tang, Z. et al. enabled the tracked combine harvester to feature three modes: straight driving, unilateral braking steering, and smooth in-situ steering without soil accumulation [29]. Ding et al. reviewed the chassis configurations and key technologies of agricultural robots by comparing wheeled, tracked, and wheel-legged platforms, and concluded that tracked and hybrid chassis require terrain-vehicle coupled modeling and multi-modal control to improve trafficability and stability in complex fields [30]. Zhang et al. systematically summarized modeling and path-tracking control methods for unmanned agricultural ground vehicles, showing that high-precision operation in unstructured fields depends on terrain-adaptive vehicle models, slip compensation, and real-time robust controllers [31]. Wang et al. designed an agricultural tractor path-tracking controller by combining an improved Stanley guidance law, extended state observation, sideslip compensation, and sliding mode heading control, and demonstrated that this strategy reduces lateral tracking error and overshoot under low-adhesion and U-turn conditions [32]. Wei et al. established a longitudinal-slip dynamic model for differential-drive agricultural vehicles and used EKF-based slip estimation together with adaptive backstepping control, concluding that slip compensation and adaptive coefficient adjustment can improve tracking accuracy and stability when trajectory curvature changes sharply [33]. Song et al. developed a rice-transplanter path-tracking method through extended line-of-sight guidance, extended state observers, and adaptive fuzzy sliding mode control, and verified that the lateral error could be stably controlled within 0.1 m in paddy-field experiments [34]. These studies provide important control-oriented references for agricultural vehicle mobility.

Current research on tracked chassis mainly focuses on attitude leveling, ride comfort, and the analysis of soil compaction. However, few studies have investigated the active regulation of the ground contact pressure of tracks. Therefore, this study developed a variable ground pressure tracked chassis driven by electric cylinders (Fig 1). This mechanism can adjust the effective ground contact length, making it possible to switch between a regular mode and a low-pressure mode. A mathematical model was derived to calculate the geometric relationship between the cylinder stroke and the linkage mechanism. Then, a 3D structural finite element model was built to analyze the load transmission and the structural stability. Finally, a prototype was manufactured and tested in a soil bin using pressure sensors to measure the soil stress distribution under different modes.

**Fig 1:**
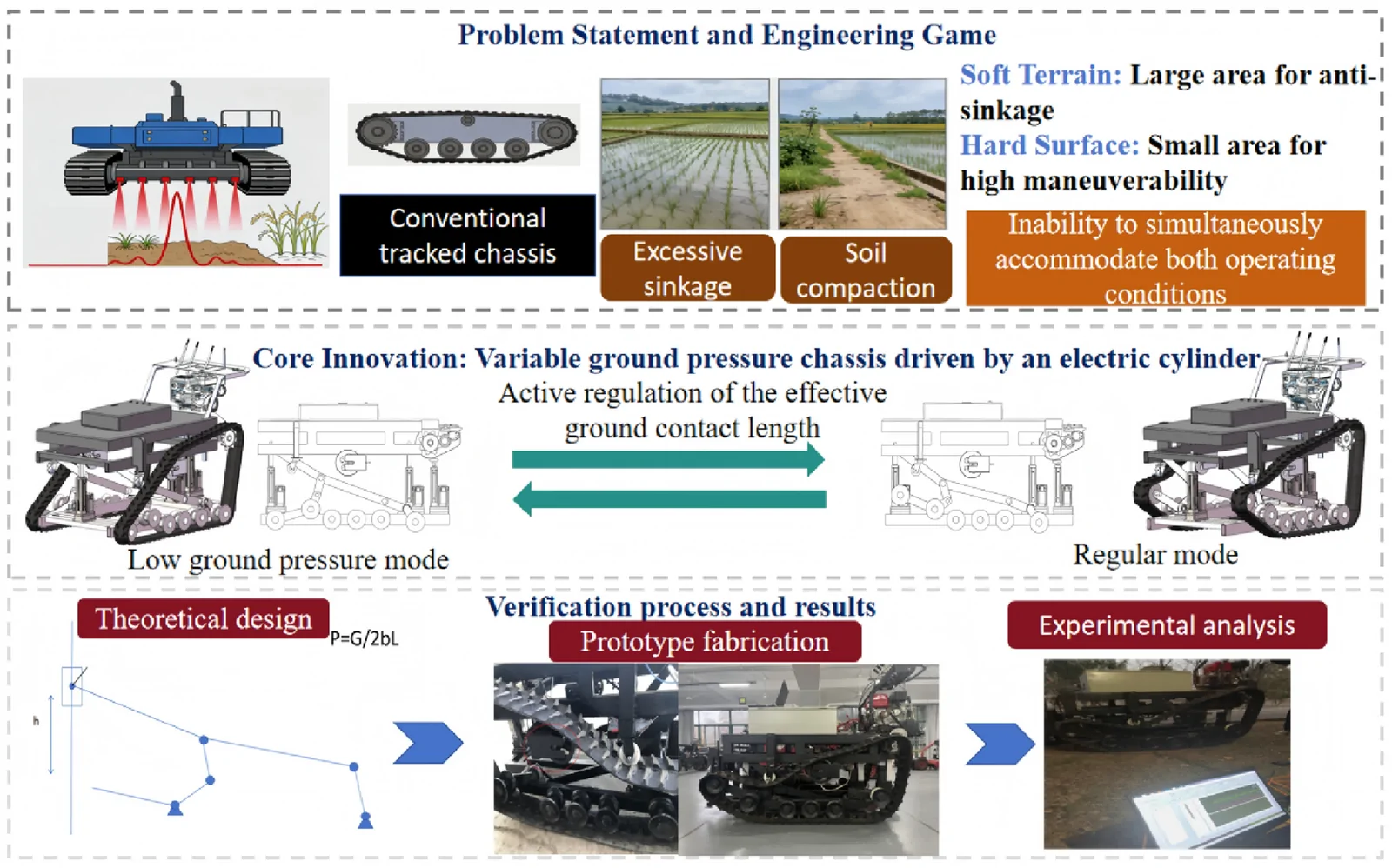
Graphical abstract.

## 2. Materials and methods

### 2.1. Mechanism design of the variable ground pressure tracked chassis

To resolve the contradiction between the passability and low-pressure protection of tracked chassis under different operational road conditions, a variable ground pressure tracked chassis device was proposed and designed in this study, as illustrated in Fig 2. In terms of configuration, this chassis is mainly composed of three core modules: a dual-layer chassis frame system, a vertical drive unit, and a multi-stage linkage assembly.

**Fig 2:**
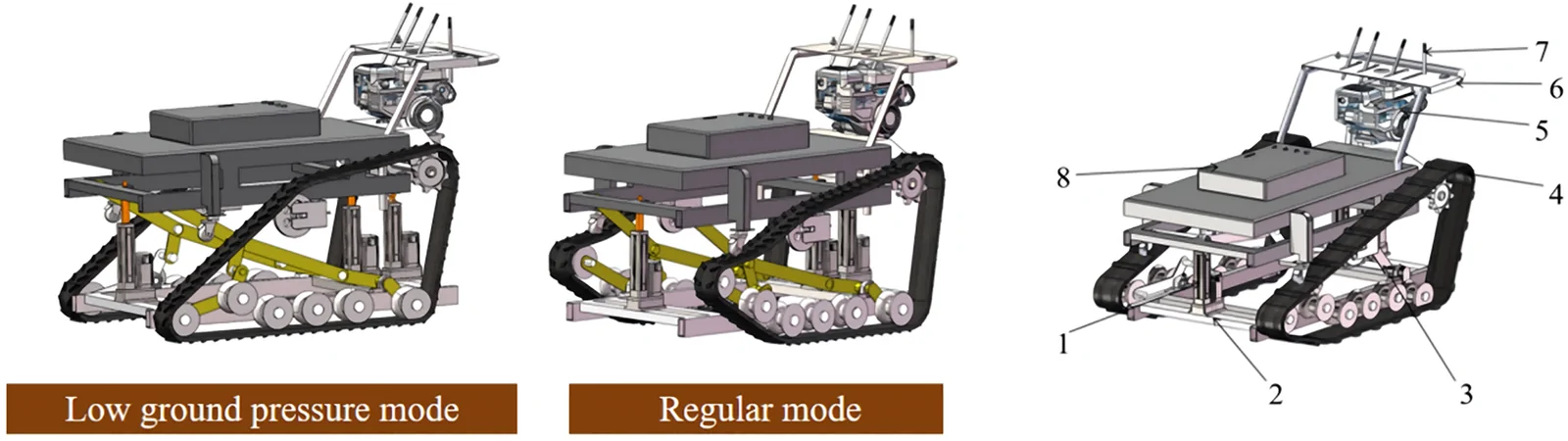
Overall structure diagram of the variable ground pressure tracked chassis. 1. Electric cylinder; 2. Lower chassis frame; 3. Linkage transmission mechanism;4. Track;5. Engine; 6. Handrail frame;7. Control lever;8. Upper floating frame.

The dual-layer chassis frame system includes a lower chassis frame and an upper floating frame, as shown in Fig 3. The lower chassis frame serves as the basic load-bearing component, while the upper floating frame acts as the floating driving component; relative displacement in the vertical direction between the two is achieved via guide columns. As the load-bearing base for whole-machine operations, the lower chassis frame is equipped with three sets of high-precision driving electric cylinders installed in a distributed manner. The power output ends of the electric cylinders are rigidly connected to the upper floating frame through reinforced bearing blocks, forming a stable three-point support lifting system, as illustrated in the figure. This structural design not only ensures that the chassis possesses a sufficient regulation stroke in the vertical degree of freedom but also guarantees the dynamic stability of the whole machine’s center of gravity during the ground contact pressure regulation process through the synchronous and coordinated action of the three electric cylinders.

**Fig 3:**
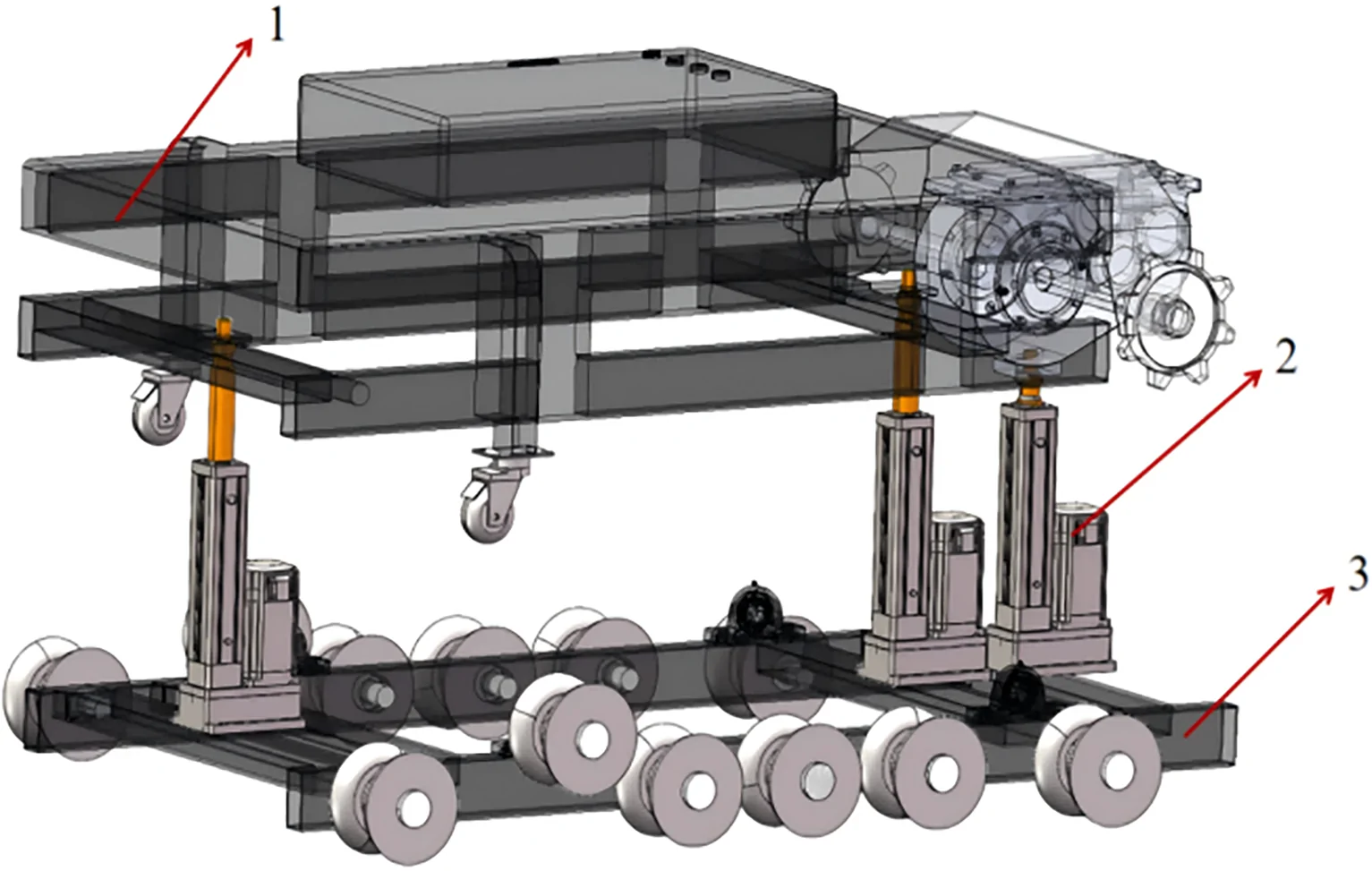
Dual-layer chassis frame system. 1. Upper floating frame;2. Electric cylinder; 3. Lower chassis frame.

The design of the linkage transmission mechanism employs a coaxial power transmission mechanism, effectively resolving the issue of motion interference within compact spaces. The core of the mechanism consists of a revolute joint composed of a linear guide shaft fixed to the chassis frame and a coaxially rotating compression bar, as shown in Fig 4. When the electric cylinders drive the upper floating frame to perform vertical lifting, the compression bar rotates synchronously, transmitting the linear driving force to the execution units located at the front and rear ends of the tracked chassis via a spatial linkage system comprising connecting rods and articulating rods.

**Fig 4:**
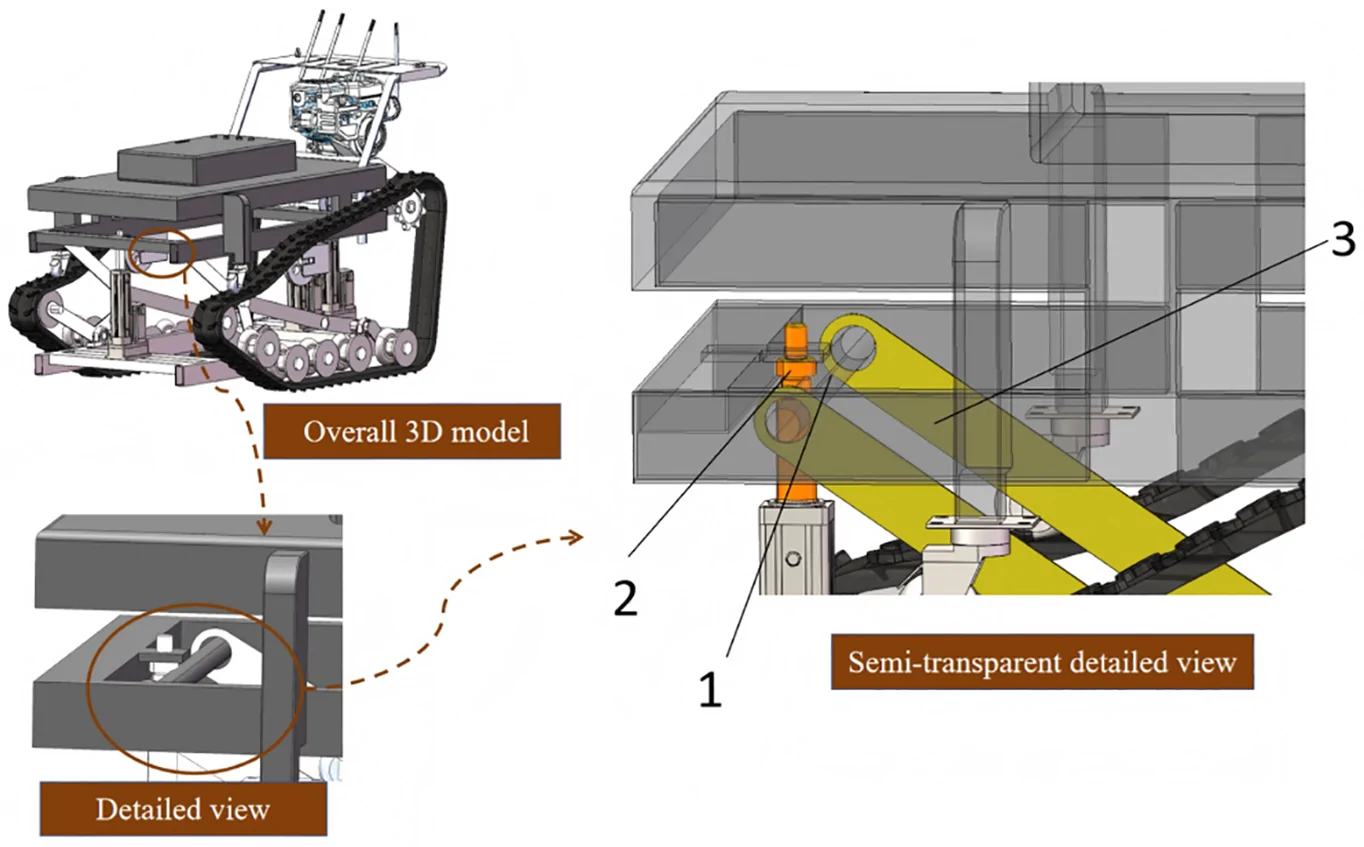
Coaxial power transmission. 1. Linear guide shaft;2. Electric cylinder lifting rod; 3. Compression bar.

Serving as the key to the morphological reconfiguration of ground contact, the execution unit adopts a lever deflection configuration based on a V-rod structure, as illustrated in Fig 5. The V-rod is hinged to the side plate of the chassis frame via a central pin shaft; one end carries the idler wheel and the fixed wheelset, while the other end is connected to the linkage system. In the conventional mode, the electric cylinders are retracted, causing the upper floating frame to descend, which in turn drives the linear guide shaft and the compression bar to move downward. Driven by the traction of the connecting rod and the articulating rod, the front V-rod and the rear rocker arm deflect upward around their respective fulcrums. At this point, the idler wheel and the rear oscillating wheel are lifted, resulting in a reduced ground contact area and increased ground contact pressure, which facilitates steering and ridge crossing.

**Fig 5:**
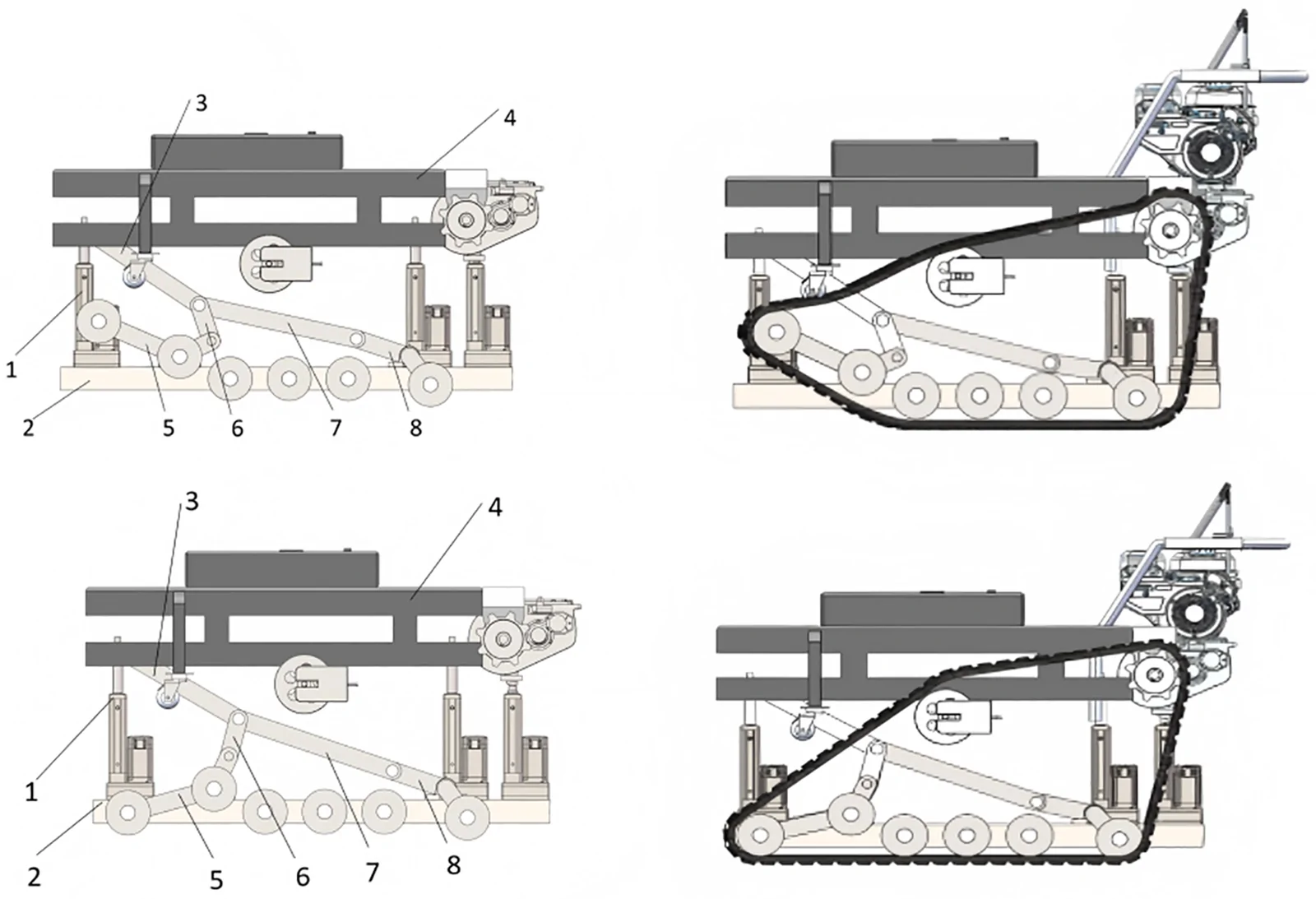
Working principle and schematic diagram of the regulation mechanism. (a) Conventional mode, (b) Low-pressure mode. 1. Electric cylinder;2. Lower chassis frame; 3. Compression bar; 4. Upper floating frame; 5. V-rod; 6. Connecting rod; 7. Articulating rod; 8. Rocker arm.

When entering soft and wet paddy fields for operation, the electric cylinders are controlled to extend, causing the upper floating frame to ascend. The linkage mechanism thereby transmits tensile force, pushing the V-rod and the rocker arm to rotate downward to a horizontal position. At this time, the full length of the track contacts the ground, the effective ground contact length reaches its maximum value, and the ground contact pressure is significantly reduced, thereby achieving anti-sinkage protection for the soil.

### 2.2. Kinematic model of the linkage regulation mechanism

The ground passability of a tracked chassis determines its sinkage on soft ground and its impact on soil structure. Ground contact pressure is the primary criterion for evaluating this passability, and its allowable limit depends on the soil bearing capacity. To control the variable ground pressure mechanism described in Section 2.1, quantitative criteria are needed to switch between the high-pressure and low-pressure modes. This study uses the closed-loop vector position equation method to analyze the kinematics of the multi-stage linkage mechanism. This method establishes the mathematical relationship between the electric cylinder stroke and the chassis ground contact state. The 3D linkage mechanism has complex geometric constraints. To simplify the calculations, secondary spatial features were removed, and key kinematic nodes were extracted. The 3D structure was then mapped into a 2D planar kinematic model, as shown in Fig 6. This 2D model keeps the main kinematic characteristics of the system and provides clear geometric boundaries for the closed-loop vector equations. Structurally, the multi-stage linkage regulation mechanism systems on the left and right sides of the chassis are symmetric along the longitudinal axis. During active adjustment, the distributed electric cylinders actuate synchronously. Because the rigid crossbeams and linear guide shafts strictly constrain the assembly, the motion of the internal linkages is confined to parallel longitudinal-vertical planes without lateral deflection. Therefore, the 3D spatial kinematics can be reliably projected onto a 2D planar model without a loss of kinematic fidelity.

**Fig 6:**
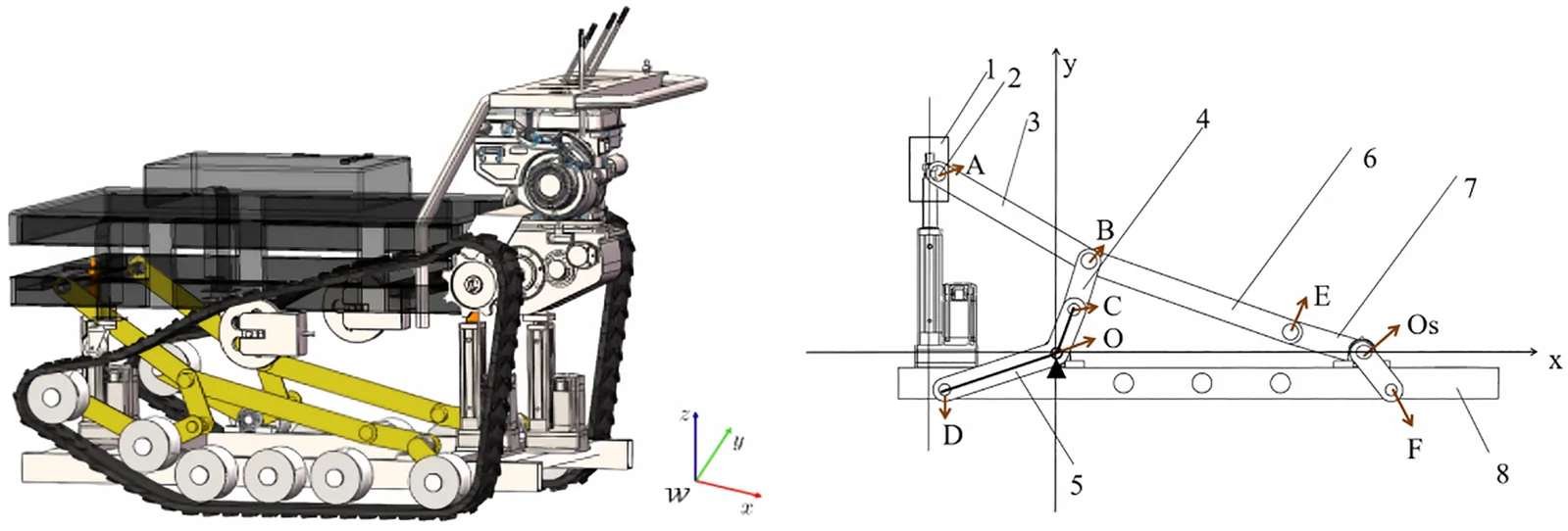
Kinematic diagram of the linkage regulation mechanism. 1. Electric cylinder; 2. Linear guide shaft; 3. Compression bar; 4. Connecting rod; 5. V-rod;6. Articulating rod;7. Rocker arm;8. Lower chassis frame.

Based on this, a parameterized geomdetric simulation model was constructed using the MATLAB platform. By specifying the input stroke of the driving electric cylinders, the position coordinates of the hinge points, the posture variations of the track frame, and the corresponding variation patterns of the final ground contact area and ground contact pressure under different strokes can be directly calculated.

The analysis results indicate that a stable monotonic mapping relationship exists between the output stroke of the driving electric cylinders and the ground contact pressure of the chassis. By solely controlling the electric cylinder stroke, the preset regulation targets for ground contact pressure can be precisely achieved, and the switching between high- and low-pressure driving modes can be accomplished. This provides a clear quantitative basis for the subsequent motion control of the chassis.

The basis for this monotonic relationship is the combined constraint of the linkage geometry and the average ground pressure model. Under the quasi-static adjustment condition, the total chassis load G and the single-track width b remain constant, and the average ground contact pressure can be expressed as $\text{P}=\frac{\text{G}}{\text{2bL}}$, where L is the effective ground contact length. The closed-loop kinematic model shows that, within the designed stroke range and without reaching a mechanical singular position, extension of the electric cylinders drives the V-rod and rocker arm downward, thereby increasing L continuously. Therefore, when $\frac{dL}{dS}$> 0, $\frac{dP}{dS}=-\frac{G}{\text{2}bL}\frac{dL}{dS}<\text{0}$, indicating that the ground contact pressure decreases monotonically with increasing cylinder stroke S. This conclusion holds under the assumptions that the three electric cylinders move synchronously, the track remains tensioned without slip or tooth skipping, the vehicle load is unchanged during mode switching, and the analysis is limited to the designed stroke range of the regulation mechanism. However, this monotonic conclusion strictly holds under the following structural and operational assumptions: (1) the three distributed electric cylinders move in perfect synchronization without tilting the upper floating frame; (2) the track remains adequately tensioned by the tensioner spring throughout the reconfiguration process, with no track slackening or tooth skipping occurring; (3) the total dynamic load of the vehicle remains unchanged during the mode switching; and (4) the kinematic operation is confined within the predefined mechanical stroke limits of the regulation mechanism.

First, based on the kinematic diagram of the linkage regulation mechanism shown in Fig 7, a planar Cartesian coordinate system is established, with the fixed rotation center of the execution unit’s V-rod on the chassis frame designated as the coordinate origin O(0,0). In this reference frame, the positive X-axis points horizontally to the right, and the positive Y-axis points vertically upward. The lower chassis frame is regarded as the stationary coordinate datum. Meanwhile, since the linear guide shaft is integrally fixed to the upper floating frame, its vertical coordinate varies in response to the extension and retraction of the driving electric cylinder. In the diagram, point C represents the hinge point between the V-rod and the connecting rod; point E is the hinge point between the rocker arm and the articulating rod; point B denotes the common hinge point of the three components (the compression bar, the connecting rod, and the articulating rod); and point A is the hinge point connecting the linear guide shaft and the compression bar. Additionally, Os denotes the fixed rotation center of the rocker arm, with coordinates (Xs, Ys). To maintain mathematical consistency in the geometric derivation of the multi-stage linkage mechanism, a standard Cartesian coordinate system with an upward-pointing Y-axis is adopted in this model.

**Fig 7:**
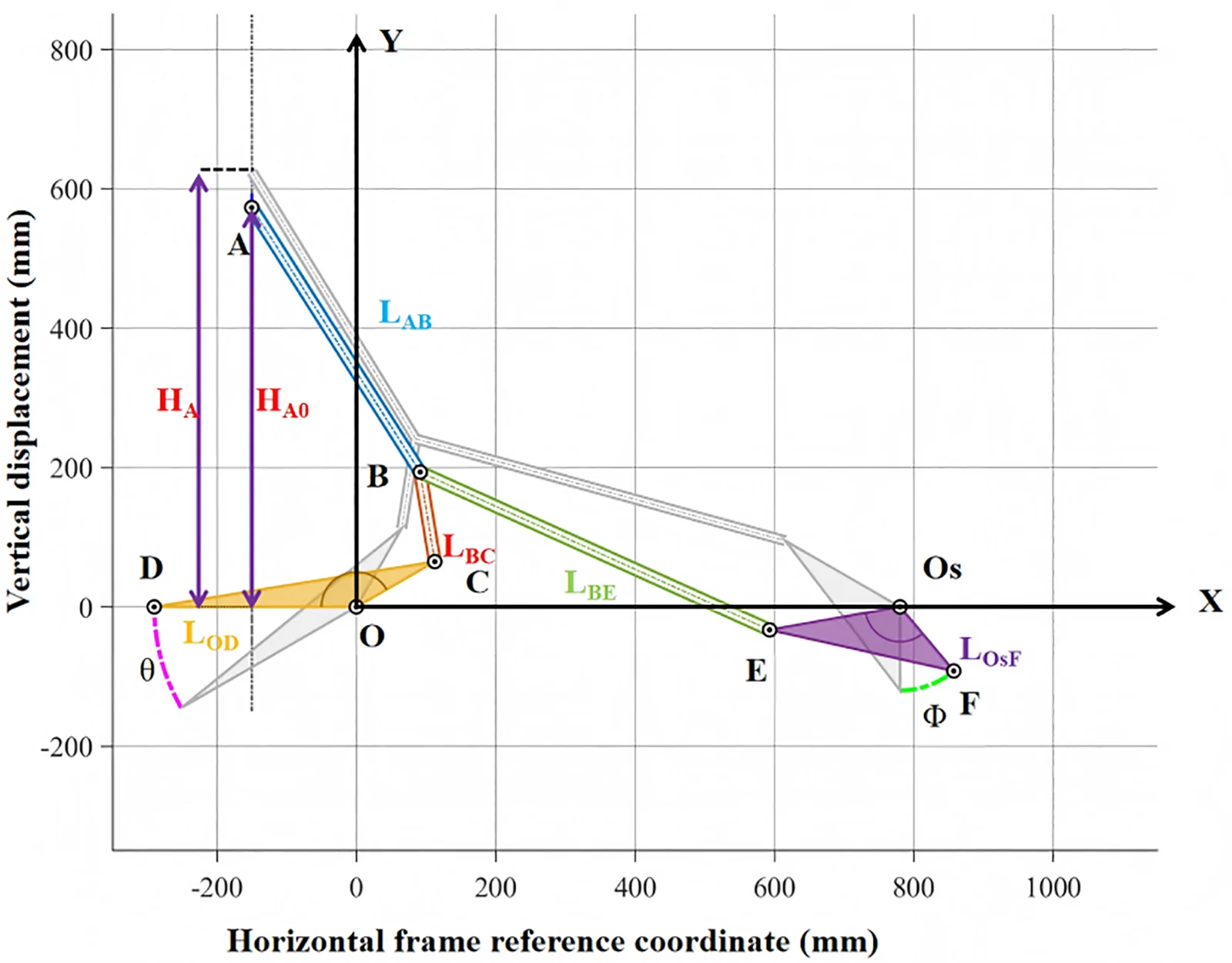
Parameterized geometric simulation model.

The V-rod rotates around the origin O, and its position is determined by the input angle. Based on Euler’s formula or trigonometric transformations, the coordinate equations for point C are:


$$\begin{array}{c} x_{c}=l_{OD}\;cos\theta \end{array}$$ (1)

$$\begin{array}{c} y_{c}=l_{OD}\;sin\theta \end{array}$$ (2)

Where ${\text{L}}_{\text{OD}}$ is the length of the V-rod end, and θ is the rotation angle of the V-rod.

The rocker arm rotates around the fixed point Os. Assuming its rotation angle is ϕ, the coordinate equations for point E are:


$$\begin{array}{c} x_{E}=x_{s}+l_{OsF}\;cos\phi \end{array}$$ (3)

$$\begin{array}{c} y_{E}=y_{s}+l_{OsF}\;sin\phi \end{array}$$ (4)

Where ${\text{L}}_{\text{OsF}}$ is the length of the rocker arm, and ϕ is the rotation angle of the rocker arm.

From the above, the end poses of the V-rod and the rocker arm are obtained.

To solve for the motion trajectory of the multi-rod intersection point B, given that hinge point B serves as the central hub for kinematic transmission within the mechanism, its position is governed by the rigid length constraints of the connecting rod CB and the articulating rod EB. Therefore, point B must simultaneously satisfy the following two conditions: it must be located on the circumference of a circle centered at C with a radius of CB By combining the coordinate equations of points C and E, a system of geometric constraint equations can be established, utilizing the two points as centers and their corresponding rod lengths as radii:


$$\begin{array}{c} (x_{B}-x_{C}{)}^{\text{2}}+(y_{B}-y_{C}{)}^{\text{2}}=l_{BC}^{\text{2}} \end{array}$$ (5)

$$\begin{array}{c} (x_{B}-x_{E}{)}^{\text{2}}+(y_{B}-y_{E}{)}^{\text{2}}=l_{BE}^{\text{2}} \end{array}$$ (6)

Where ${\text{L}}_{\text{BC}}$ is the length of the connecting rod, and ${\text{L}}_{\text{BE}}$ is the length of the articulating rod.

By applying the elimination method, the linear equation is obtained:


$$\begin{array}{c} \text{2}x_{B}(x_{C}-x_{E})+\text{2}y_{B}(y_{C}-y_{E})=l_{BE}^{\text{2}}-l_{BC}^{\text{2}}-x_{E}^{\text{2}}-y_{E}^{\text{2}}+x_{C}^{\text{2}}+y_{C}^{\text{2}} \end{array}$$ (7)

By defining ${\text{A}}_{\text{0}}=\text{2}({\text{x}}_{\text{C}}-{\text{x}}_{\text{E}})\;,{\text{B}}_{\text{0}}=\text{2}({\text{y}}_{\text{C}}-{\text{y}}_{\text{E}}),{\text{C}}_{\text{0}}={\text{l}}_{\text{BE}}^{\text{2}}-{\text{l}}_{\text{BC}}^{\text{2}}-{\text{x}}_{\text{E}}^{\text{2}}-{\text{y}}_{\text{E}}^{\text{2}}+{\text{x}}_{\text{C}}^{\text{2}}+{\text{y}}_{\text{C}}^{\text{2}}$

It simplifies to:


$$\begin{array}{c} A_{\text{0}}x_{B}+B_{\text{0}}y_{B}=C_{\text{0}} \end{array}$$ (8)

Substituting Equation (8) into Equations (5) and (6) yields a quadratic equation with respect to ${\text{x}}_{\text{B}}$:


$$\begin{array}{c} Mx_{B}^{\text{2}}+Nx_{B}+P=\text{0} \end{array}$$ (9)

Where M,N and P are all coefficients.

By applying the quadratic formula to solve for ${\text{x}}_{\text{B}}$, and substituting the result into Equation (8), ${\text{y}}_{\text{B}}$ can be obtained:


$$\begin{array}{c} x_{B}=\frac{-N\pm \sqrt{N^{\text{2}}-\text{4}MP}}{\text{2}M} \end{array}$$ (10)

Thus, the coordinates of point B are determined. Since the linear guide shaft of the electric cylinder is fixed vertically, its horizontal coordinate remains constant. Given that the coordinates of point A are (${\text{x}}_{\text{A}}$, HA), and the coordinates of point B are (${\text{x}}_{\text{B}}$, yB), the length of the compression bar AB establishes the following geometric constraint:


$$\begin{array}{c} |AB|=\sqrt{(x_{B}-x_{A}{)}^{\text{2}}+(y_{B}-h{)}^{\text{2}}}=l_{AB} \end{array}$$ (11)

$$\begin{array}{c} (x_{A}-x_{B}{)}^{\text{2}}+(H_{A}-y_{B}{)}^{\text{2}}=l_{AB}^{\text{2}} \end{array}$$ (12)

Given that the electric cylinder is arranged vertically from top to bottom, the absolute height of the linear guide shaft node must always be greater than that of the lower node B. By solving the equation and discarding the negative root, the absolute height of the electric cylinder’s linear guide shaft is obtained as follows:


$$\begin{array}{c} H_{A}=y_{B}+\sqrt{l_{AB}^{\text{2}}-(x_{A}-x_{B}{)}^{\text{2}}} \end{array}$$ (13)

Considering that the electric cylinder slider is positioned above the mechanism in the actual engineering layout, an inverse transformation is applied to Equation (11) to derive the functional relationship between the electric cylinder strokeSand the intermediate variables(xB,yB):


$$\begin{array}{c} S=H_{A}-H_{A\text{0}}=y_{B}+\sqrt{l_{AB}^{\text{2}}-(x_{A}-x_{B}{)}^{\text{2}}}-H_{A\text{0}} \end{array}$$ (14)

Therefore, Equation (14) expresses the electric cylinder strokeSas a nonlinear function of the V-rod angle and the rocker arm angle.

The kinematic analysis shows that the regulation mechanism has highly nonlinear transmission characteristics. The mathematical model proves the feasibility of regulating the ground contact pressure. It also calculates the exact relationship between the driving unit and the execution unit.

The displacement input of the electric cylinder is transmitted through the multi-bar mechanism, ultimately manifesting as changes in the spatial coordinates of the front guide wheel D and the rear fixed wheel F. The projection coordinate equations for the front wheel center point D and the rear wheel center point F on the horizontal X-axis are respectively:


$$\begin{array}{c} X_{D}=X_{O}+l_{OD}\;cos(\theta +\text{1}\text{5}{\text{0}}^{\circ }) \end{array}$$ (15)

$$\begin{array}{c} X_{F}=X_{Os}+l_{OsF}\;cos(\phi +\text{1}\text{2}{\text{0}}^{\circ }) \end{array}$$ (16)

Since the bearing capacity of a tracked chassis on soft terrain is primarily provided by its horizontal ground contact section, its real-time effective ground contact length Lcan be characterized by the horizontal span between the front and rear wheels:


$$\begin{array}{c} L(\theta ,\phi )=X_{F}-X_{D}=X_{O}s+l_{OsF}\;cos(\phi +\text{1}\text{2}{\text{0}}^{\circ })-l_{OD}cos(\theta +\text{1}\text{5}{\text{0}}^{\circ }) \end{array}$$ (17)

According to classical terramechanics theory, the core criterion for the ground contact pressure distribution of tracked vehicles is:


$$\begin{array}{c} P=\frac{G}{\text{2}bL} \end{array}$$ (18)

Where P is the real-time ground contact pressure of the track (kPa), G is the total gravity load of the device (N), b s the ground contact width of a single rubber track (m), and L is the derived real-time effective ground contact length (m).

By substituting the implicit function relationship of the electric cylinder established in Equation (12) and Equation (17) into Equation (18), the mapping model between the electric cylinder displacement and the ground contact pressure can be constructed:


$$\begin{array}{c} P(h)=\frac{G}{\text{2}b\cdot [X_{Os}+l_{OsF}\;cos(\phi (h)+\text{1}\text{2}{\text{0}}^{\circ })-l_{OD}\;cos(\theta (h)+\text{1}\text{5}{\text{0}}^{\circ })]} \end{array}$$ (19)

### 2.3. Structural finite element analysis of the variable ground pressure chassis

To verify the structural stability of the variable ground pressure tracked chassis under different operational postures, this section conducts a comparative static simulation analysis of the chassis under self-weight loading in two typical working modes: the regular mode and the low ground pressure mode. By applying standard gravitational acceleration and corresponding ground support boundary conditions, the total deformation and equivalent stress distribution contours for both modes were solved and extracted. The regular mode generally corresponds to the operating condition where the electric cylinders are retracted, and the effective ground contact length of the track is relatively short. Conversely, the low ground pressure mode corresponds to the condition where the electric cylinders are extended, the multi-stage linkage mechanism is fully deployed, and the effective ground contact length reaches its maximum. The static simulation results of the chassis under these two modes are illustrated in Fig 8.

**Fig 8:**
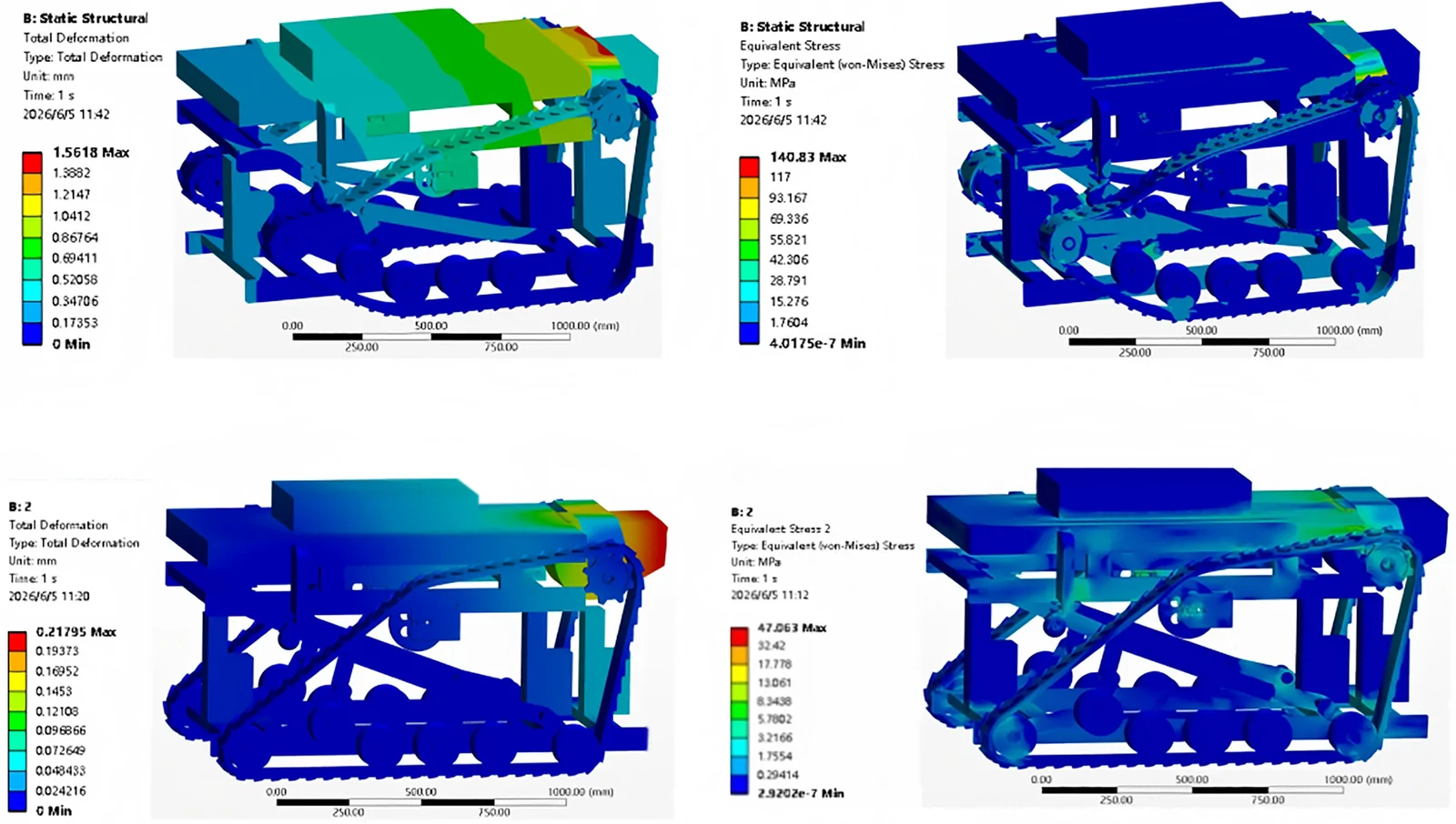
Contours of total deformation and equivalent stress in regular and low ground pressure modes. (a) Total deformation and equivalent stress distribution contours in regular mode, (b) Total deformation and equivalent stress distribution contours in low ground pressure mode.

To improve reproducibility, the finite element modeling procedure was specified in detail. The 3D model was imported into the finite element solver, and small holes, fillets, and other local features that did not affect the global load path were simplified. The frame, linkage members, brackets, and pins were assigned Q235 structural steel properties (elastic modulus$\;\text{E}=\text{2}.\text{06}\times \text{1}{\text{0}}^{\wedge }\text{5MPa}$, Poisson’s ratio nu = 0.30, density rho = 7850 kg/m^3^, and yield strength sigmay = 235 MPa). The rubber track was modeled as a deformable elastic body, while the ground was represented as a supporting contact surface for comparative static loading. The model was meshed with tetrahedral elements. A global element size of 10 mm was used for the main frame, and local refinements of 3–5 mm were applied around pin holes, welded transitions, wheel-track contact zones, and the track-ground interface. Frictional contact was defined between the track and the ground support, and bonded contact was used for welded or rigidly connected structural parts. The lower surface of the ground support was constrained in the vertical direction, while the chassis load was introduced through gravitational acceleration and the self-weight of all structural components. This boundary condition allowed the two regulation postures to be compared under identical load transfer conditions.

As shown in the total deformation and equivalent stress contours (a), the maximum displacement of the frame in the regular mode occurs at the installation point between the rear end cover and the driving wheel, with the maximum deformation reaching 1.5618 mm. Due to the shortened ground contact length, a pronounced cantilever beam effect is formed at the rear of the frame, resulting in a relatively large downward deflection. The maximum equivalent stress in this mode is 140.83 MPa. The stress concentration region primarily appears at the welded transition between the front suspension bracket and the main frame. Although this maximum stress value of 140.83 MPa remains below the 235 MPa yield limit of Q235 steel—providing a safety factor of approximately 1.64 without plastic yielding—this stress concentration area is prone to becoming a weak point for fatigue failure under long-term alternating loads.

In the low ground pressure mode, the maximum total deformation of the chassis drops sharply to 0.2179 mm. Due to the deployment of the multi-stage linkage mechanism, the front road wheels move forward, effectively extending the foundation support span for the frame. This significantly mitigates the cantilever beam effect at the front end, thereby greatly improving the overall stiffness performance of the frame. The maximum equivalent stress in this mode is significantly reduced to 47.063 MPa. As observed from the equivalent stress contour distribution (b), because the multi-stage linkage mechanism exerts an excellent load-homogenizing effect, the overall structural load on the frame is more uniformly distributed, leaving most areas in a low-stress state. Consequently, the stress concentration phenomenon at the front bracket is fundamentally alleviated.

To investigate the mechanical behavior of the chassis tracks pressing against the ground, the normal stress contours at the contact interface between the track plates and the ground foundation were specifically extracted for comparative analysis. Finite element contact analysis techniques were utilized to deeply explore the static ground contact pressure distribution characteristics of the chassis under the two typical operational modes. In the finite element structural mechanics solution system, the sign of the normal stress explicitly characterizes the loading attributes of the material, where a positive value indicates localized tensile stress and a negative value denotes compressive stress. Therefore, a smaller algebraic value represents a higher concentration of compressive stress.

The surface normal stress distribution contour of the track contact interface in the regular mode is illustrated in Fig 9. In this mode, the electric cylinders are retracted, resulting in a relatively shortened effective ground contact length. Consequently, the total vehicle load is highly concentrated directly beneath the load-bearing wheels in the middle section of the chassis. As observed from the color gradient distribution of the contour, the contact interface stress exhibits distinctly discrete and non-uniform characteristics. Directly extracting the contact surface nodal data reveals that the localized maximum surface ground compressive stress in the regular mode rapidly escalates to 1.5 MPa. When the chassis is switched to the low ground pressure mode, the corresponding surface normal stress distribution contour is shown in Fig 10. In this mode, the electric cylinders are fully extended, and the multi-stage linkage mechanism is completely deployed, maximizing the effective ground contact area of the track. It can be clearly observed from the surface normal stress contour that the original localized high-pressure regions are significantly alleviated. The entire track contact interface presents a uniform, low-stress distribution state, with the maximum surface ground compressive stress attenuating to 0.05 MPa.

**Fig 9:**
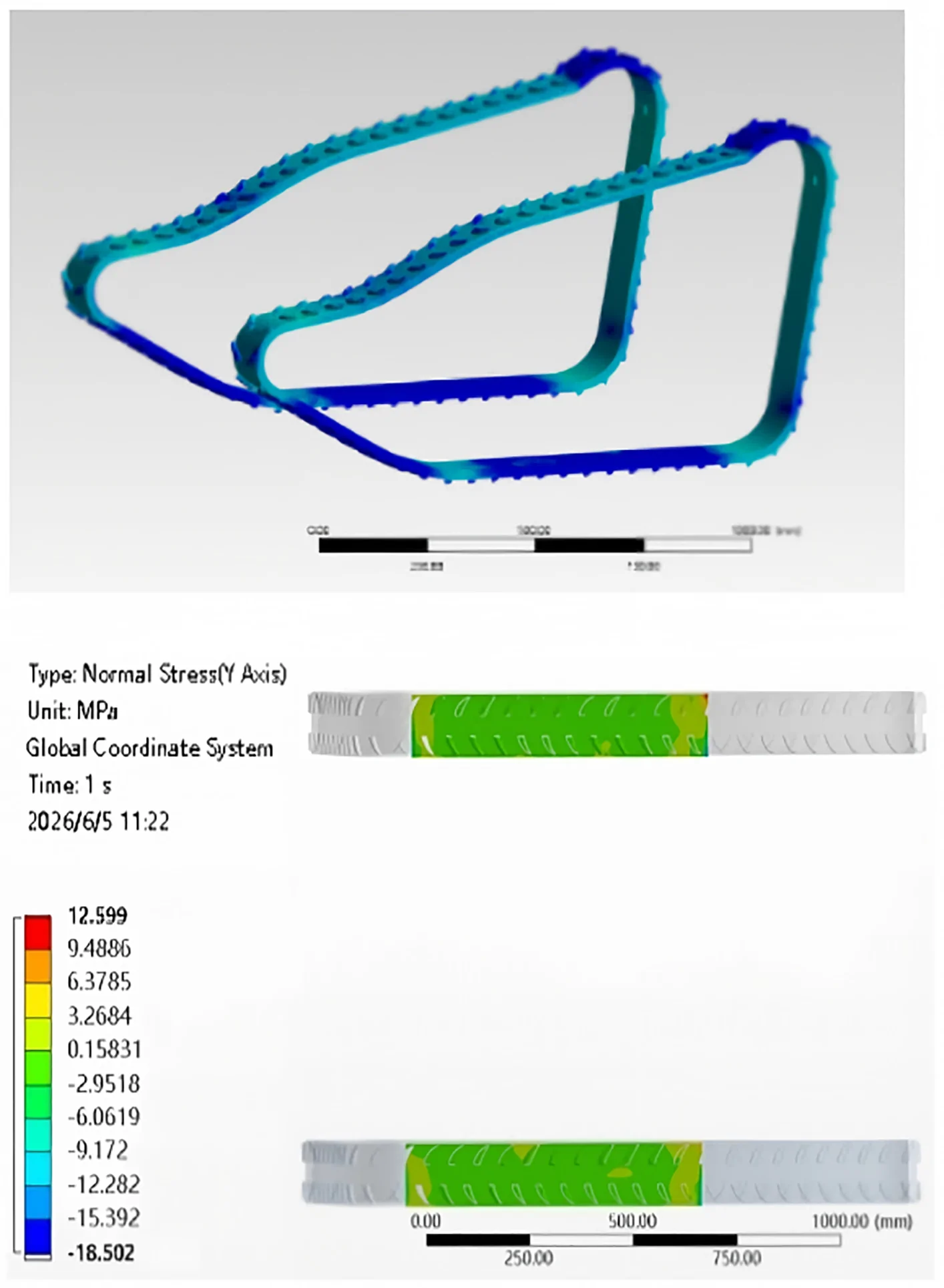
Normal stress distribution contour of the track in regular mode.

**Fig 10:**
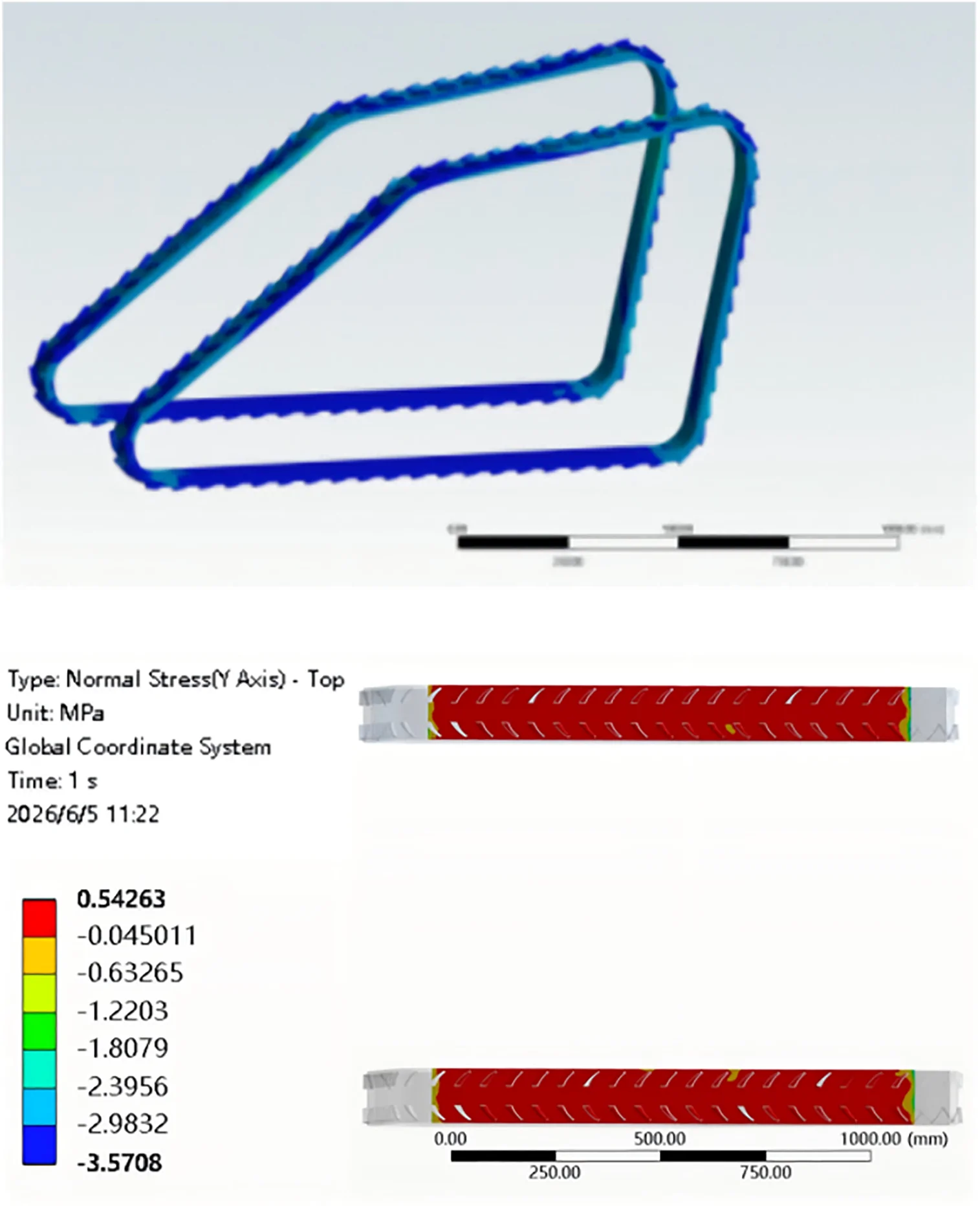
Normal stress distribution contour of the track in low ground pressure mode.

However, a detailed analysis of the contact interface shows that the outer surface of the track has anti-slip lugs. When these lugs contact the simplified ground surface in the FEA model, local stress concentrations occur. These numerical errors affect the accuracy of the macroscopic load distribution analysis. Therefore, the contact pressure directly measured from the outer surface is much higher than the actual ground pressure. To reduce the numerical error caused by the track lugs, this study used an equivalent ground pressure evaluation method based on the internal load transfer path (Fig 11). The vehicle’s weight is transmitted through the load-bearing wheels to the flat inner surface of the track. Therefore, a longitudinal data path was set along the bottom centerline of the track’s inner surface. As shown in Fig 12, the normal stress along this inner path shows a wave-like pattern, with the peaks matching the locations of the load-bearing wheels. The results show that in the regular mode, the mean compressive stress on the inner track is about 0.03 MPa. In the low-pressure mode, the linkage mechanism distributes the load more evenly across all wheels. As a result, the mean compressive stress decreases to 0.008 MPa.

**Fig 11:**
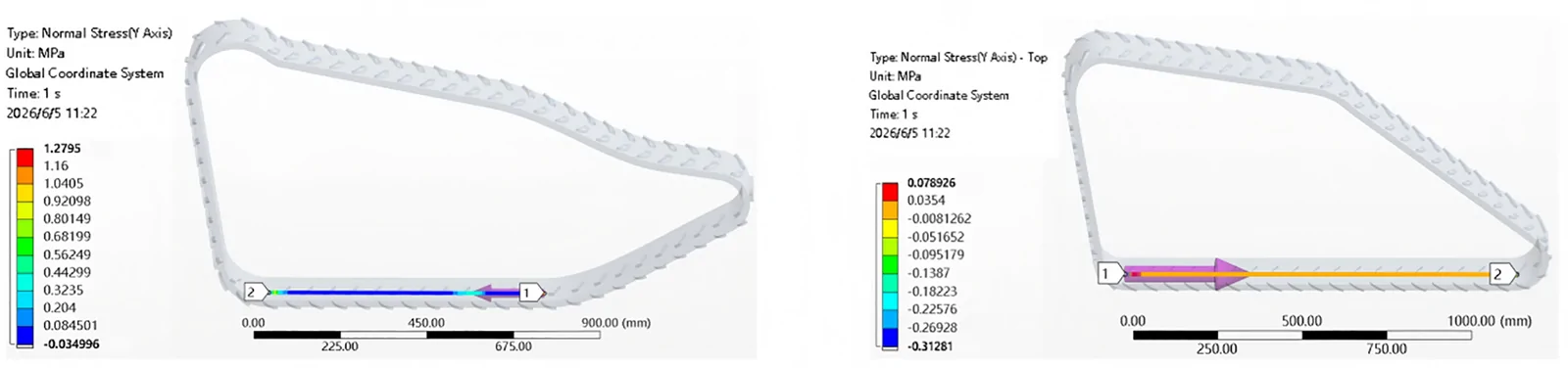
Internal load transfer path of the track.

**Fig 12:**
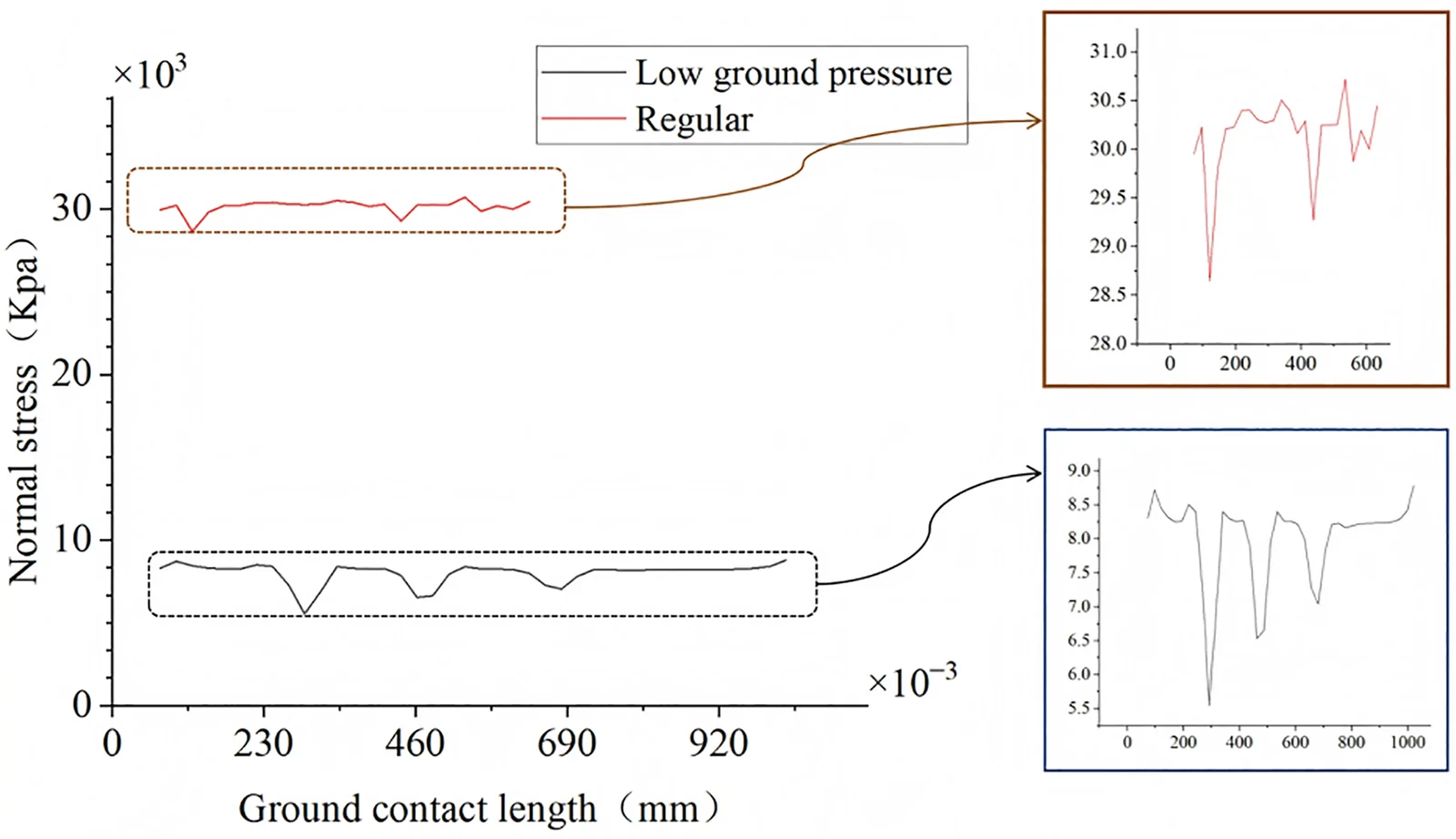
Normal stress distribution curve along the inner track surface.

To validate the reliability of the aforementioned internal load transfer path method, the FEA stress results were correlated with classical terramechanics analytical solutions. According to the average ground pressure model, the theoretical pressures for the regular and low ground pressure modes are 20.96 kPa and 14.06 kPa, respectively. In comparison, the FEA internal path method yielded localized mean compressive stresses of approximately 0.03 MPa (30 kPa) and 0.008 MPa (8 kPa). The numerical deviations between the two methods arise because the theoretical formula assumes an ideal, uniform pressure distribution, whereas the FEA realistically captures the localized peak-and-valley stress concentrations beneath the discrete load-bearing track rollers. Despite these absolute numerical differences, both approaches demonstrate a highly consistent and substantial stress-reduction trend. Furthermore, this numerical stress-attenuation trend is macroscopically corroborated by the direct dynamic soil bin measurements (where actual soil peak stress decreased from 93 kPa to 63 kPa) presented in Section 3.2, thereby confirming the methodological validity of the FEA approach.

The macroscopic data derived from this inner path highly correlate with the theoretical estimations of the ground contact pressure for the physical prototype presented in Section 3.1, thereby validating the scientific rigor of the post-processing correction scheme.

### 2.4. Prototype fabrication and experimental setup

Based on the structural design of the key components detailed in Section 2.2, the complete machine was fabricated. The manufactured variable ground contact pressure tracked chassis prototype, as shown in Fig 13, primarily consists of a power drive module, a chassis travel module, an electrical control module, and a regulation mechanism drive module. The machine adopts a modular integrated design, with overall dimensions of 2100 mm in length, 1200 mm in width, and 1600 mm in height. Its total weight is approximately 400 kg, and it has a designed load capacity of 250 kg. The key parameters of the prototype are shown in Table 1.

**Fig 13:**
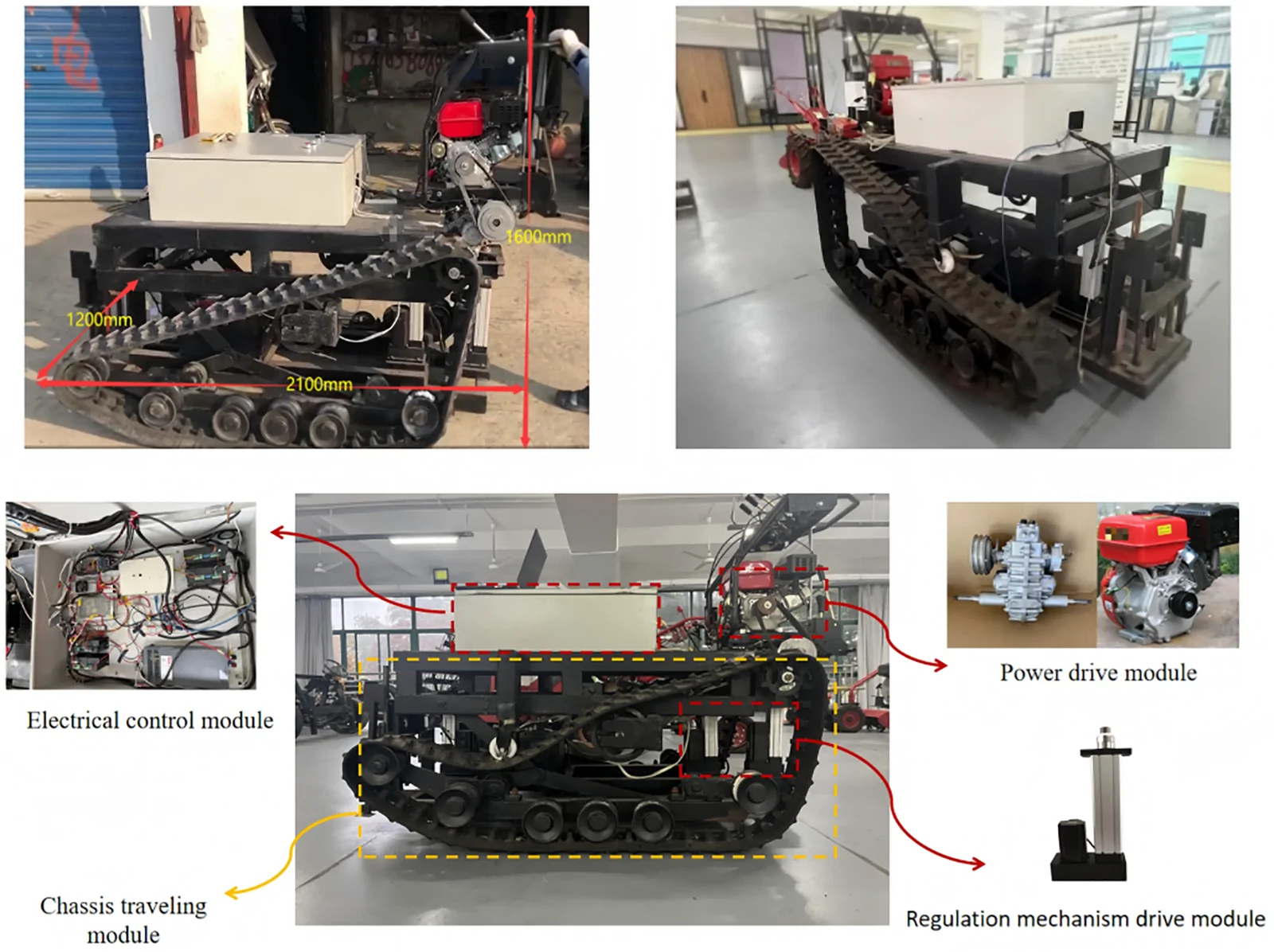
Prototype of the variable ground contact pressure tracked chassis.

**Table 1:** Key parameters of the prototype.

Key Parameter	Value
**Overall mass**	400 kg
**Overall dimensions (L-W-H)**	2100mm × 1200 mm × 1600 mm
**Minimum ground clearance**	>200mm
**Ground contact length adjustment range**	550mm∼820 mm
**Ground pressure adjustment range**	±20%

Regarding the control strategy for mode switching, a parallel position-control method is currently implemented within the electrical control module. Since the morphological reconfiguration of the chassis is primarily performed while the vehicle is stationary, a central microcontroller simultaneously dispatches identical pulse-train commands to the independent servo drivers of the three distributed electric cylinders. Because these cylinders possess identical electromechanical specifications and their vertical displacements are strictly coupled by the rigid upper floating frame and linear guide shafts, this parallel command approach ensures sufficient macroscopic synchronization. It allows the multi-stage linkage mechanism to complete the frame ascent or descent smoothly without mechanical binding.

A dynamic traveling load experiment was conducted to evaluate the soil compaction characteristics of the chassis. The primary test variables included the regular mode, the low ground pressure mode, and three traveling speeds at 0.5 m/s, 1.0 m/s, and 1.2 m/s. Figs 14a and 14b show the sensor layout. The measurement depth was set to 15 cm below the soil surface. Five miniature soil pressure sensors with a 28 mm diameter and a 0.2 MPa measurement range were fixed onto a 3D-printed rigid plate. The sensor array was moved to a new undisturbed position after each test pass to prevent cumulative soil compaction. The horizontal center-to-center spacing between adjacent sensors was 5 mm, creating a total detection width of 200 mm. Fig 14d shows that this configuration completely covers the core load-bearing area of a single track to accurately capture the peak stress values.

**Fig 14:**
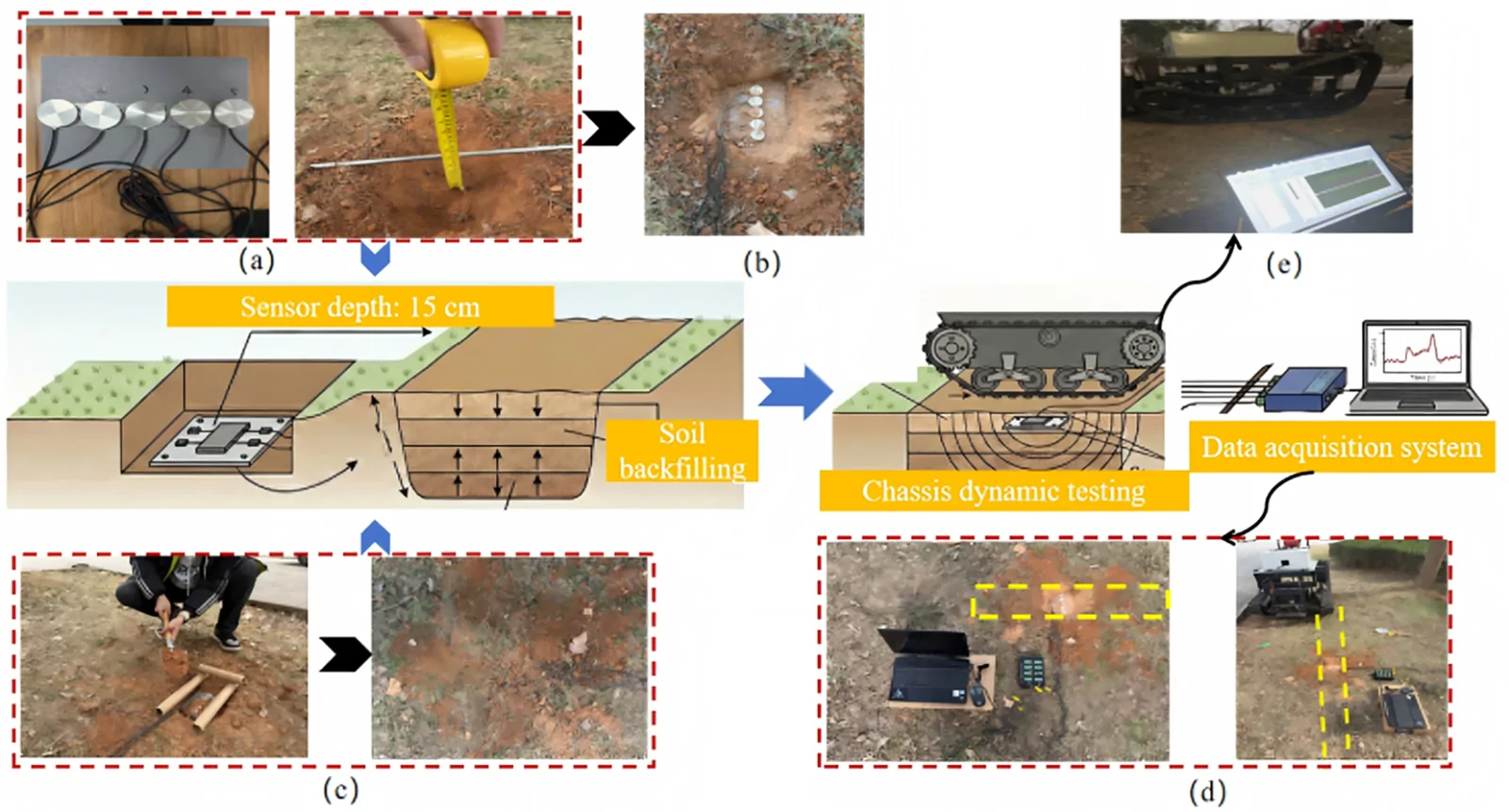
Experimental implementation process. (a) 3D-printed rigid plate and soil trench depth; (b) Sensor placement; (c) Trench excavation and soil backfilling using a custom guide template and flat hoe; (d) Experimental site setup; (e) Chassis testing.

The experimental procedure was conducted in strict accordance with soil mechanics testing standards. As illustrated in Fig 14(c), a test trench with a length of 20 cm and a depth of approximately 15 cm was first excavated in the field using a custom-made guide template and a flat-bottom hoe. Subsequently, the sensor mounting plate was positioned, and the soil was backfilled and compacted in layers in an effort to restore the compactness of the undisturbed soil. To ensure experimental reproducibility and clarify the baseline conditions, the fundamental physical and mechanical properties of the remolded soil used in the test trench were evaluated. The test environment primarily consisted of 7.5%low-moisture soil, characterized by a particle density of 1450 kg/m^3^, a Poisson’s ratio of 0.25, and a shear modulus of 6 × 10^6 Pa. As shown in Fig 14(e), during the passes of the chassis across the test area, the sampling frequency of the data acquisition system was set to 1000 Hz to accurately capture the dynamic stress waveforms generated as the chassis traveled over the sensors under various speed conditions. The parameters of the soil pressure sensors and the data acquisition system are shown in the Table 2 below. The choice of this single, specific depth is justified by both agronomic importance and soil mechanics principles. From an agronomic perspective, 15 cm corresponds to the core zone of the cultivated plow layer in typical paddy fields, where crop root systems predominantly proliferate and where topsoil compaction causes the most severe and detrimental impacts on root growth and nutrient absorption. From a terramechanics standpoint, the dynamic loading and additional vertical stress transmitted from tracked agricultural vehicles typically manifest as highly concentrated peak pulses within this shallow subsoil layer before dissipating into deeper strata or pan layers. Therefore, monitoring the dynamic stress at a depth of 15 cm serves as the most sensitive and practically meaningful indicator to evaluate and compare the soil-protecting effectiveness of the chassis under different driving modes.

**Table 2:** Key parameters of instruments and data acquisition system.

Instrument	Model	Specifications and Parameters
Miniature soil pressure sensor	BWM	Measurement range: 0.01kPa-200 kPa
		Diameter:28 mm
		Overload capacity:150%
		Accuracy error: 0.3 F S
Dynamic signal testing and analysis system	YBY-801	Number of channels:8
		A/D resolution:24-bit
		Strain measurement range: ± 19999με
		Maximum resolution:1με

## 3. Results and discussion

### 3.1. Evaluation of regulation performance and key component reliability for the variable ground contact pressure mechanism

First, the chassis power was turned on and the control software was connected. In the current prototype, the switching between the two modes is manually triggered by the operator via the software interface based on terrain conditions. To ensure operational safety and track tensioning stability, the morphological switching is strictly performed while the vehicle is stationary, avoiding sudden center-of-gravity shifts during active travel. All electric cylinders were checked to ensure they were at their zero positions with proper track tension. Then, the regular mode test was performed. A frame descent command was sent via the software, causing the electric cylinders to retract synchronously. When the mechanism reached its limit and stopped, the ground contact points at the front and rear were marked. A steel tape measure was used to measure the effective ground contact length of the single-side track. This value was recorded as Lmin and averaged over three measurements (Table 3). After that, the low ground pressure mode test was conducted. A frame ascent command was issued, causing the electric cylinders to extend synchronously and lift the frame. The track rollers pressed down on the track. Once the track was completely flat and the mechanism was locked, the effective ground contact length was measured using the same method. This value was recorded as Lmax and averaged over three measurements. During the entire process, the tensioner spring mechanism was observed to ensure the track remained taut.

**Table 3:** Recorded data of measured ground contact length.

Test Condition	Measurement No.	Left-side length (cm)	Right-side length (cm)	Average length (cm)
Regular mode	1	55.2	55.0	55.1
	2	54.9	55.1	
	3	55.0	54.9	
Low ground pressure mode	1	82.1	81.9	82
	2	82.0	82.2	
	3	81.8	82.0	

As illustrated in Fig 15, in the regular mode, the chassis exhibits a profile with both ends upturned, and the ground contact points are concentrated in the middle section of the chassis; in the low ground pressure mode, the chassis assumes a completely flat configuration, resulting in a significant increase in the ground contact area.

**Fig 15:**
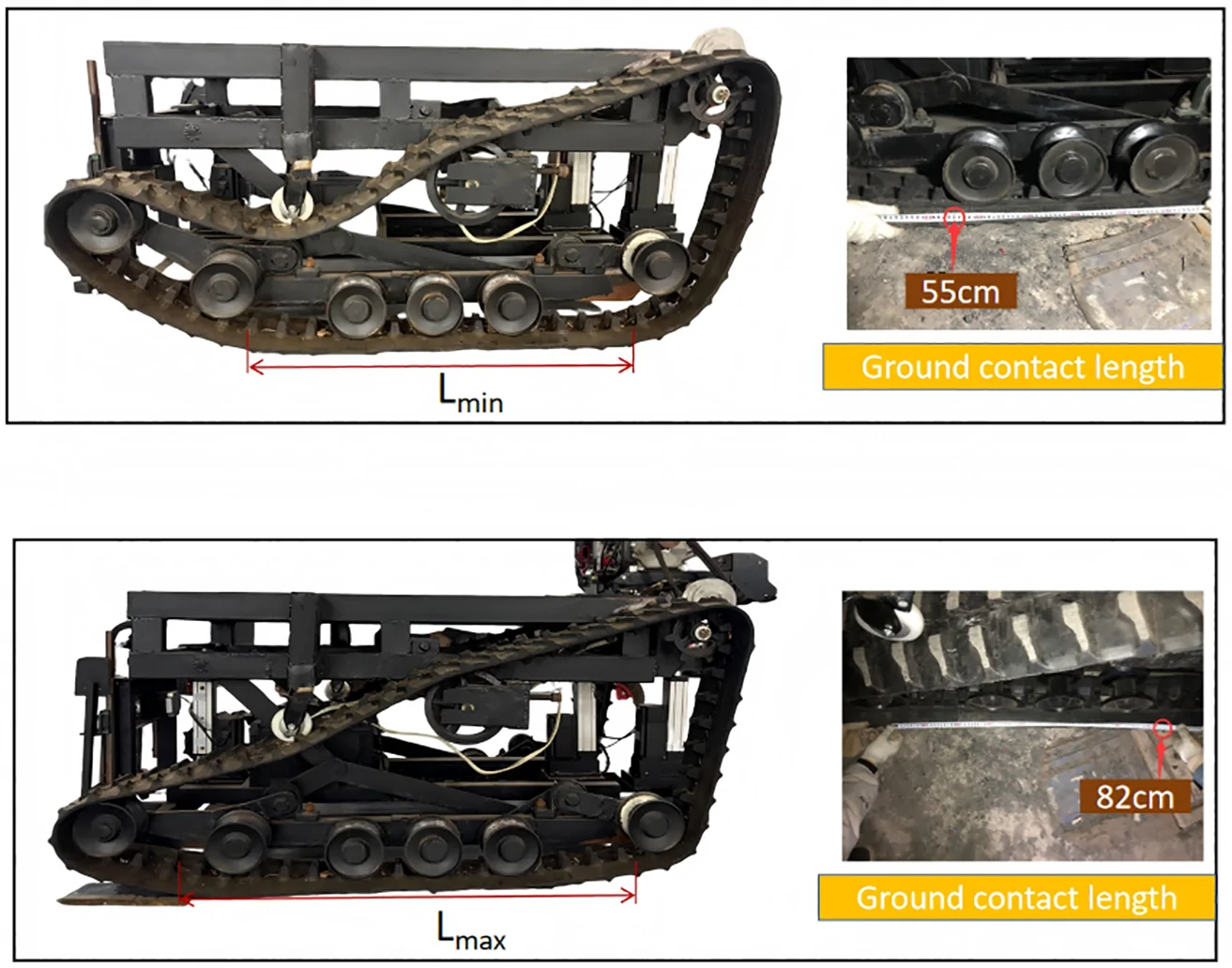
Measurement of ground contact length under different modes. (a) Measurement of ground contact length in regular mode, (b) Measurement of ground contact length in low ground pressure mode.

Furthermore, these physical measurements directly validate the accuracy of the 2D kinematic simplification. The actual measured effective ground contact lengths—averaging 55.1 cm in the regular mode and 82 cm after the frame ascent in the low ground pressure mode—highly correlate with the theoretical displacement boundaries calculated from the 2D planar model. This strong experimental correlation confirms that the simplified 2D mathematical model accurately captures the macroscopic motion behavior of the actual 3D linkage regulation mechanism.

According to vehicle terramechanics theory, the formula for the average ground contact pressure is $\text{P}=\frac{\text{G}}{\text{2bL}}$, where the total vehicle weight is 3920 N, the single track width is 0.17 m, and L represents the measured effective ground contact length. First, the numerical calculation of the ground contact pressure was conducted. In the regular mode, the measured ground contact length Lmin = 0.55m; substituting this into the formula yields a maximum ground contact pressure Pmax = 20.96kPa. In this mode, the higher ground contact pressure assists in cutting through soil surface roots when traveling on hard ground, while the shorter ground contact length significantly reduces the frictional resistance torque during steering, thereby enhancing steering flexibility. Conversely, in the low ground pressure mode, the measured ground contact length Lmax = 0.82m, resulting in a calculated minimum ground contact pressure Pmin = 14.06kPa. This value is generally lower than the yield limit of deep-mud fields, which effectively prevents the chassis from sinking and ensures high trafficability in soft-soil environments.

The regulation capability of the prototype can be evaluated by the total variation rate of the ground contact length $\eta_{\text{L}}$, which is expressed as $\eta_{\text{L}}=\frac{{\text{L}}_{\text{max}}-{\text{L}}_{\text{min}}}{{\text{L}}_{\text{max}}}$, and calculated to be approximately 32.9%. Using the median of the regulation range as a baseline, the dynamic regulation amplitude δ of the ground contact pressure of the prototype is ± 19.7%. The experimental results demonstrate that the prototype achieves the regulation capability of the design target (±20%), and the ground contact pressure is continuously adjustable within the range of 14.06–20.96 kPa.

During the experiment, it was observed that as the frame transitioned from its lowest to its highest position, the track tensioner retracted accordingly. This action effectively absorbed the excess track length resulting from the change in geometric configuration. Throughout the entire process, neither track slackening nor tooth skipping occurred.

As shown in Fig 16, testing was conducted on various surfaces to simulate high-frequency transport operations, such as turning and U-turns on both hard and soft grounds, aiming to observe the stability performance of the tracks. During the physical traveling tests on hard paved surfaces, the chassis, when switched to the regular mode, exhibited excellent maneuverability. Due to the shortened effective ground contact length, the lateral frictional resistance between the tracks and the ground was significantly reduced. Consequently, the prototype demonstrated rapid response and smooth motion during straight-line travel and in-situ steering. Throughout the travel process, the tracks exhibited no significant jamming or abnormal vibration. Under soft ground conditions, when the chassis was operating in the low ground pressure protection mode, the entire machine demonstrated outstanding trafficability. The full-length ground contact of the tracks effectively dispersed the dynamic loads. During uniform forward propulsion, the prototype maintained a stable traveling posture with continuous power output, and no obvious track slippage was observed.

**Fig 16:**
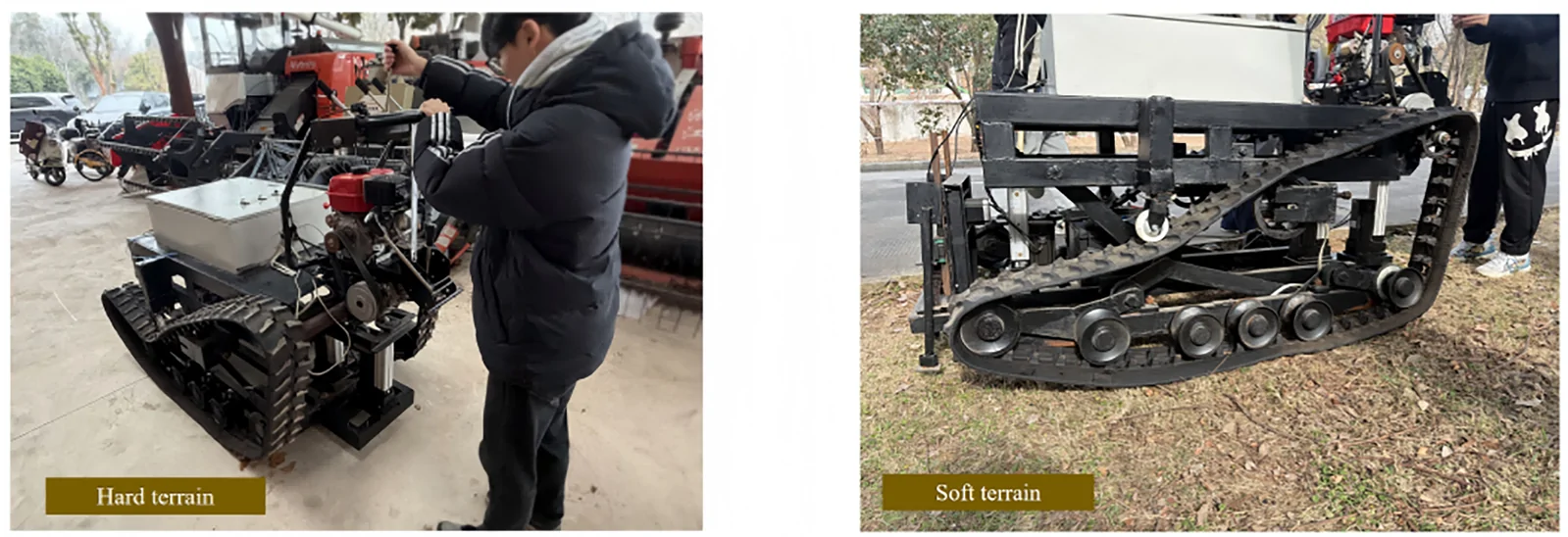
Testing under different ground conditions. (a) Hard paved surface, (b) Grass surface.

### 3.2. Evolution of chassis dynamic driving characteristics under variable operating conditions

The chassis performance was evaluated using multi-dimensional indicators by selecting two different working modes (the regular mode and the low ground pressure mode) and utilizing three different traveling speeds (0.5 m/s, 1.0 m/s, and 1.2 m/s) as the primary influencing factors.

As shown in Fig 17 and Table 4, the transverse stress distributions of the two modes are different. In the regular mode, the vertical load is concentrated on one side of the track due to the shorter contact length. Fig 17(b) shows that the local peak stress on a single side reaches about 175 kPa. This indicates that the regular mode is prone to uneven load transfer on soft ground.

**Fig 17:**
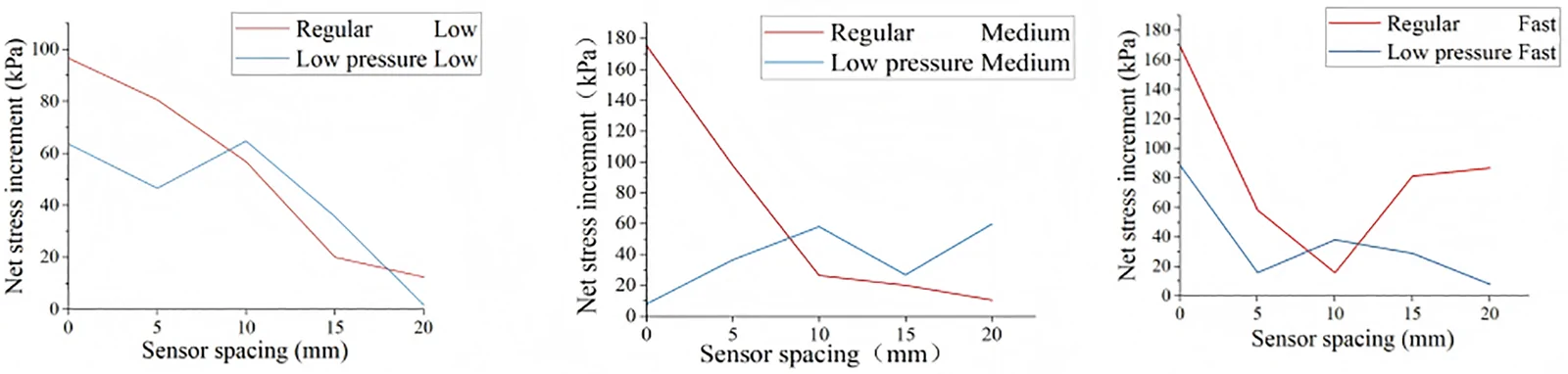
Net stress increments at different speeds under regular and low ground pressure modes. (a) Net stress increment at low speed under regular and low ground pressure modes. (b) Net stress increment at medium speed under regular and low ground pressure modes. (c) Net stress increment at high speed under regular and low ground pressure modes.

**Table 4:** Average transverse net stress increment at different measurement points under various working conditions.

Traveling speed	Regulation mode	0mm	5mm	10mm	15mm	20mm
Low	Regular	98.2	80.5	58.1	20.3	12.5
	Low pressure	64.0	46.5	65.1	36.2	2.1
Medium	Regular	175.0	98.6	26.3	20.1	10.4
	Low pressure	8.5	37.2	58	26.8	60.5
High	Regular	170.5	58.4	15.6	81.2	86.5
	Low pressure	88.3	15.8	38.4	28.6	8.2

Note: The values in the table represent the highest net stress peaks during the dynamic testing process. These data have been processed by deducting the initial static soil pressure at a depth of 15 cm and applying Gaussian filtering for noise reduction, representing the maximum transient additional pressure experienced at the measurement point at the exact moment the vehicle passes.

In the low-pressure mode, the contact area increases, leading to a more uniform transverse stress distribution. As shown in Fig 17(c), the maximum peak stress decreases from 175 kPa in the regular mode to about 65 kPa, achieving a reduction of approximately 63%. The larger contact area helps distribute the load across the track width.

To analyze the contact mechanics of the chassis on soft terrain, 3D surface plots were created to show the normal contact stress distribution over time and transverse position. Fig 18(a), 18(b), and 18(c) show that the ground contact stress in the regular mode is discontinuous and concentrated at all three speeds. Due to the limited contact area, the dynamic load forms narrow and steep stress peaks. Furthermore, the loading and unloading times are short, resulting in rapid stress changes. These high-amplitude stress pulses can cause local soil shear failure and excessive sinkage.

**Fig 18:**
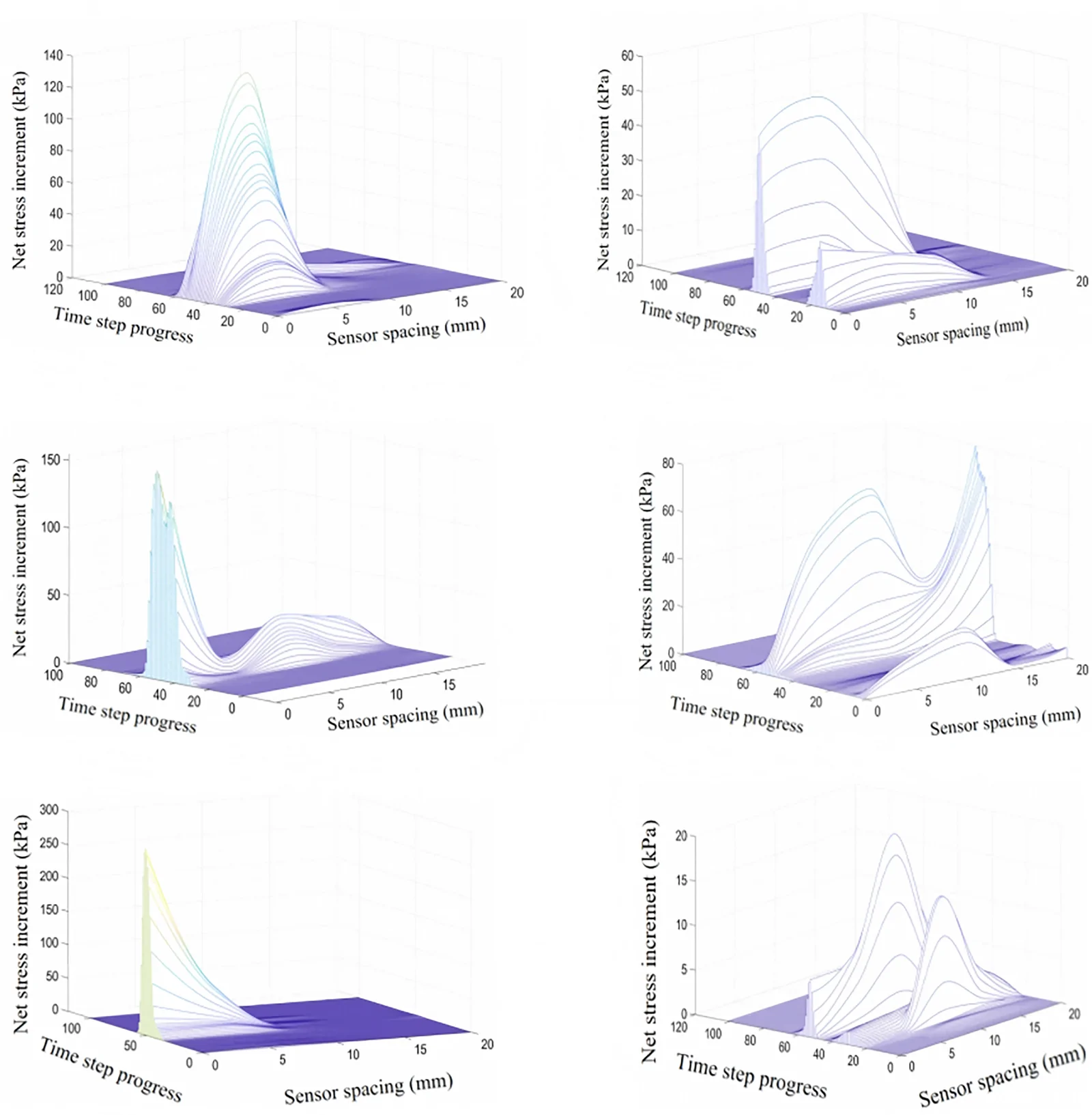
Waterfall plots of normal contact stress increment under regular and low ground pressure modes at different speeds. (a) Comparison at low speed: Regular mode, Low speed, Low ground pressure mode, Low speed. (b) Comparison at medium speed: Regular mode, Medium speed, Low ground pressure mode, Medium speed. (c) Comparison at high speed: Regular mode, High speed, Low ground pressure mode, High speed.

In the low ground pressure mode, the shape of the 3D stress surface changes significantly. The isolated stress peaks are reduced, and the stress is distributed more evenly along both the transverse and time axes. This indicates that the increased contact area effectively distributes the vertical load across the track width. Additionally, the load application time is extended, which reduces transient impact loads. The chassis-soil interaction changes from a concentrated stress state to a more uniform load-bearing state. Comparing the results across the three speeds, the low ground pressure mode maintains a uniform stress distribution at different velocities. It effectively avoids the sharp stress increases seen in the regular mode at higher speeds, showing stable mechanical performance.

To evaluate how the ground contact area affects dynamic vertical stress, time history curves for the conventional and low-pressure modes at slow, medium, and fast speeds were extracted (Fig 19). In the conventional mode (Figs 19a, 19c, and 19e), the stress curves showed sharp, multi-peak fluctuations at all speeds. Because the effective contact area is small in this mode, the vehicle’s weight and dynamic loads were not evenly distributed, causing severe vertical stress concentration. Specifically, at slow speed (Fig 19a), the limited contact area caused high stress pulses in the central region, reaching a peak of about 93 kPa. At medium speed (Fig 19c), the stress concentration moved toward the edges, and the peak stress increased to approximately 108 kPa. At fast speed (Fig 19e), the stress curves showed alternating pulses with a maximum peak of about 52 kPa. These results indicate that a smaller ground contact area amplifies local dynamic loads during driving. In the low-pressure mode (Figs 19b, 19d, and 19f), the effective ground contact area increased, which changed the shape of the stress curves. The stress peaks were reduced, and the curves became flatter and broader. This shows that the larger contact area distributes the vertical loads more evenly and reduces local pressure extremes. The test data confirmed this stress reduction: at slow speed, the peak stress decreased from 93 kPa in the conventional mode to about 63 kPa; at medium speed, it dropped from 108 kPa to approximately 38 kPa; and at fast speed, it decreased from 52 kPa to about 23 kPa. The time history data also show that when the vehicle left the test zone (unloading phase), the vertical stress in both modes returned to zero. This means the soil recovered elastically without significant deep residual compaction. During the loading phase, however, increasing the contact area effectively distributed the stress and reduced the peak values. The larger track-soil interface lowered the local stress concentrations in both spatial and temporal dimensions. This mechanism changes concentrated dynamic loads into steady, distributed loads, keeping the peak vertical stress within a safe range. These findings confirm the importance of expanding the ground contact area to reduce soil damage in agricultural operations.

**Fig 19:**
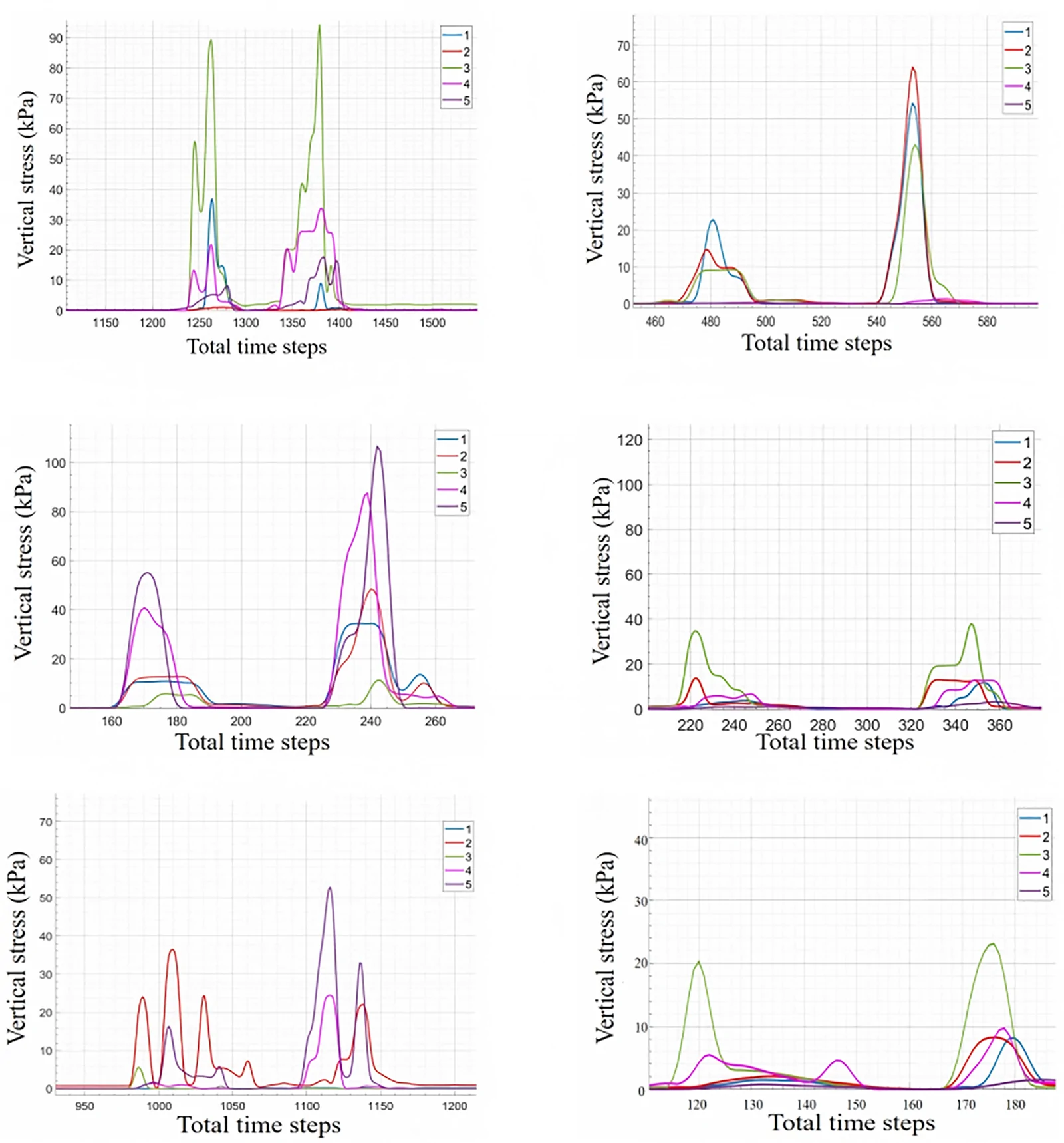
Vertical stress under regular and low-pressure modes at different speeds. (a) Vertical stress in regular slow-speed mode, (b) Vertical stress in low-pressure slow-speed mode, (c) Vertical stress in regular medium-speed mode, (d) Vertical stress in low-pressure medium-speed mode, (e) Vertical stress in regular high-speed mode, (f) Vertical stress in low-pressure high-speed mode.

Based on the experimental data, the physical prototype verifies the feasibility of the active variable ground pressure mechanism. However, this study mainly focused on testing the mechanical principles and soil responses on flat terrain. The active change in chassis geometry causes the center of gravity (CoG) to shift. This CoG shift and the vehicle’s posture changes on uneven slopes have not yet been evaluated in our current models or field tests. Future research will build a multi-body dynamics (MBD) model to analyze the dynamic CoG shift. In addition, a posture compensation algorithm using multi-sensor fusion will be developed to improve the stability and performance of the tracked chassis in complex agricultural and forestry terrains.

## 4. Conclusions

The primary contribution of this study is the development and validation of an actively controlled variable ground pressure tracked chassis. Unlike conventional tracked agricultural machinery that relies on a fixed ground contact geometry, our prototype utilizes a multi-stage linkage mechanism driven by distributed electric cylinders to dynamically adjust its effective ground contact length. The soil bin experimental results demonstrated that by expanding the contact length from 55 cm to 82 cm, the proposed low-pressure mode successfully attenuated the maximum dynamic subsoil stress at the 15 cm plow layer from 93 kPa to 63 kPa at low speeds. This represents a significant stress reduction of approximately 32%, directly verifying the mechanical feasibility of our active load-homogenizing design.

When placed in the broader context of existing literature, this variable pressure approach addresses a critical engineering bottleneck. Recent advances in agricultural chassis design have predominantly focused on omnidirectional attitude leveling or active suspension control for ride comfort. While these innovations excel at maintaining vehicle stability on hilly terrains, they do not fundamentally alter the track-soil contact area, leaving the issue of high localized soil compaction unresolved in soft paddies. By integrating spatial kinematic reconfiguration with intelligent chassis control, our proposed system bridges this gap, effectively overcoming the traditional trade-off between sinkage prevention on soft terrain and steering maneuverability on hard pavements. The practical implications of this study are particularly significant for sustainable agricultural mechanization. Repeated traffic by heavy agricultural machinery is a primary driver of subsoil compaction, which irreversibly damages the physical structure of the cultivated plow layer (typically 15–20 cm deep) and restricts crop root penetration. By actively adapting the ground contact pressure to the terrain, the proposed chassis can deploy the low-pressure mode in sensitive or highly compressible paddy fields to minimize structural soil damage, and switch back to the regular mode on hard transport roads to reduce steering resistance and power consumption. Ultimately, this adaptive morphological design provides a viable hardware foundation for the next generation of eco-friendly, low-compaction agricultural machinery.

While this study validates the fundamental mechanical feasibility and static soil-protecting performance of the proposed variable ground pressure chassis, further research is necessary to optimize its dynamic capabilities in complex field environments. Future work will primarily focus on three key areas. First, we plan to develop a comprehensive Multi-Body Dynamics and Discrete Element Method (MBD-DEM) co-simulation framework to evaluate the transient non-linearities of the chassis under full-load and continuous dynamic steering conditions. Second, to further investigate terramechanic interactions, advanced sensor matrices will be utilized to conduct multi-depth 3D soil stress profiling, providing a complete characterization of the subsoil stress distribution. Finally, to transition the prototype from stationary manual switching to fully automatic dynamic reconfiguration on the move, we will develop robust closed-loop synchronous control algorithms. By integrating state-of-the-art adaptive posture control frameworks, we aim to enable intelligent, real-time morphological adaptation under uneven agricultural loads.
